# Supplementary material for: Can implementation failure or intervention failure explain the result of the 3D multimorbidity trial in general practice: mixed-methods process evaluation
Source: BMJ Open. 2019 Nov 6;9(11):e031438. doi: 10.1136/bmjopen-2019-031438 (PMC6858134; doi:10.1136/bmjopen-2019-031438)
Supplement: Supplementary data [file bmjopen-2019-031438supp001.pdf]

Appendix 1: Tidier checklist for the 3D intervention

Additional information can be found in the published full report of the trial: Salisbury C, Man M-S, Chaplin K, Mann C, Bower P, Brookes S, et al. A patient-centred intervention to improve the management of multimorbidity in general practice: the 3D RCT. Health Serv Deliv Res 2019;7(5)

Item No	Item		Summary information and location of full detail in report
**Brief name**			
1	Provide the name or a phrase that describes the intervention	✔	Improving the management of multimorbidity in general practice – the 3D study
**Why**			
2	Describe any rationale, theory, or goal of the elements essential to the intervention	✔	Underlying theoretical basis is the Patient-centred Care Model. Intervention designed to address problems experienced by people with multimorbidity and aimed to achieve improved quality of life.<br>*Report Pages 3, 9*
**What**			
3	Materials: Describe any physical or informational materials used in the intervention, including those provided to participants or used in intervention delivery or in training of intervention providers. Provide information on where the materials can be accessed (such as online appendix, URL)	✔	An purpose-designed IT template was used within Egton Medical Information Systems (EMIS) which when completed generated a patient agenda and a patient health plan. Intervention patients received a 3D card which identified them to practices and specified their named GP.<br>*Report Pages 11-15 and Appendices 3, 5-8*<br>*Report Supplementary Material 1 and 2*
4	Procedures: Describe each of the procedures, activities, and/or processes used in the intervention, including any enabling or support activities	✔	This was highly complex intervention that incorporated:<br>Installing the EMIS template<br>Identifying and recruiting the target group<br>Allocating a named GP and nurse for each participant and issuing a 3D card to each participant to improve continuity of care.<br>Training the practice staff and clinicians<br>Organising and delivering 6 monthly 3D comprehensive reviews of all health conditions and of psychosocial factors that were delivered in 2 parts, first with the named nurse, second with the named GP.<br>Medication review by pharmacist viewing patient record remotely<br>Meetings of practice champions<br>Provision of monthly monitoring feedback to practices about their delivery of the intervention<br>*Report pages 10 -15*

Item No	Item		Summary information and location of full detail in report
**Who provided**			
5	For each category of intervention provider (such as psychologist, nursing assistant), describe their expertise, background, and any specific training given	✔	Intervention providers included GPs, nurses in general practice, pharmacists, general practice administrators and receptionists, and one secondary care physician for each area.<br>*Report page 12*
**How**			
6	Describe the modes of delivery (such as face to face or by some other mechanism, such as internet or telephone) of the intervention and whether it was provided individually or in a group	✔	Face-to-face delivery of comprehensive 6 monthly reviews. Remote performance of medication review element<br>*Report pages 11-15*
**Where**			
7	Describe the type(s) of location(s) where the intervention occurred, including any necessary infrastructure or relevant features	✔	The intervention occurred in individual general practices in three areas of the UK
**When and How Much**			
8	Describe the number of times the intervention was delivered and over what period of time including the number of sessions, their schedule, and their duration, intensity, or dose	✔	The intervention two-part reviews were delivered twice in 12 months. The intervention components were mainly delivered in these reviews carried out in nurse appointments of 30-50 minutes and in GP appointments of 20<br>*Report pages 12-13*
**Tailoring**			
9	If the intervention was planned to be personalised, titrated or adapted, then describe what, why, when, and how	✔	Practices were allowed some flexibility in how intervention delivery was organised<br>*Report page 14*
**Modifications**			
10	If the intervention was modified during the course of the study, describe the changes (what, why, when, and how)		The intervention was modified after piloting from a whole practice service change intervention to selected patients only.<br>*Report page 16 and Appendix 14*
**How well**			
11	Planned: If intervention adherence or fidelity was assessed, describe how and by whom, and if any strategies were used to maintain or improve fidelity, describe them	✔	Mixed methods were used involving both quantitative and qualitative researchers in the trial team. Quantitative methods involved electronic monitoring of delivery of intervention components. Qualitative

Item No	Item		Summary information and location of full detail in report
			methods included interviewing participants and providers and observing delivery. Strategies to maintain and improve fidelity were the monthly electronic monitoring feedback, meetings of practice champions and financial incentives<br>*Report pages 31-33*
12	Actual: If intervention adherence or fidelity was assessed, describe the extent to which the intervention was delivered as planned	✔	Half the participants received the full intended number of reviews. In delivered reviews most components were delivered but the way they were delivered varied. This is presented and discussed in the conclusion of the present paper.<br>*Report pages 77-86*
