# Supplementary material for: Can implementation failure or intervention failure explain the result of the 3D multimorbidity trial in general practice: mixed-methods process evaluation
Source: BMJ Open. 2019 Nov 6;9(11):e031438. doi: 10.1136/bmjopen-2019-031438 (PMC6858134; doi:10.1136/bmjopen-2019-031438)
Supplement: Supplementary data [file bmjopen-2019-031438supp002.pdf]

Appendix 2: Interview topic guides and observation checklist

Topic guide for lead administrator pre-implementation

What do you think of the 3D intervention?

Please can you explain how you are organising the appointments and recall for 3D

How is it similar to what you already do or plan to do?

How is it different from what you already do or plan to do?

What did you think of the information you have been given?

How might it have been improved?

How do you think it might affect the practice?

Difficulties - What will be the main challenges?

Benefits

Roles of doctors, nurses and reception staff

How do you think it might affect your work?

Difficulties – what concerns do you have?

Benefits

How do you think it might change the patients' experience?

How might it affect different types of patient?

Is there anything else you would like to say?

Topic guide for lead nurse or lead GP pre-implementation

What do you think of the 3D intervention?

How is it similar to what you already do or plan to do?

How is it different from what you already do or plan to do?

What did you think of the training for 3D?

How might it have been improved?

How do you think it might affect your practice?

Difficulties - What will be the main challenges?

Benefits

Roles of doctors, nurses and reception staff

How do you think it might affect your work?

Difficulties – what concerns do you have?

Benefits

How do you think it might change the patients' experience?

How might it affect different types of patient?

Is there anything else you would like to say?

Topic guide for follow-on interviews after consultation observation – GP or nurse

How do you feel the consultation went?

What, if anything, would you have done differently and if so why?

What went particularly well?

How did the timing go?

How happy were you with how it was structured?

How easy was it to integrate use of the template?

How easy was it to get a complete picture of the patient's concerns?

How do you feel the patient responded?

What had you planned to talk about and/or what did you want to agree a plan for?

Was anything not covered that you had wanted to talk about?

GP only

How much did you use the nurse's agenda and how helpful was it?

How helpful was the medication review?

Were you happy with the plan? Do you think the patient was happy with the plan?

Was there anything that surprised you?

Topic guide for follow-on interviews after consultation observation – patients

How do you feel the consultation went?

What went particularly well or what did you particularly like?

What, if anything, were you not happy about? *(template, timing, any particular questions)*

How well do you feel the nurse understood what you were concerned about?

How well do you feel the doctor understood what you were concerned about?

What had you planned to talk about and what did you want to agree a plan for before you went into the appointment?

Was anything not covered that you had wanted to talk about?

What do you think you will discuss with the doctor? or What plan have you come away with?

Was there anything that surprised you?

How happy were you with the amount of time you had?

How conscious were you of the computer? Did it interfere with the discussion with the doctor/nurse

Topic guide for administrator post-implementation

What is your opinion of the intervention?

What perceived benefits, downsides, and unintended consequences both positive and negative?

How has it affected the management of LTCs?

How do you think patients have responded? Which patients do you think have benefitted most?

How difficult has it been to arrange appointments and to manage the searches etc?

What has your process been?

What helped the process?

What would have made it easier?

What elements of 3D do you think would be worth continuing?

Topic guide for GPs or nurses post-implementation

1. Response to intervention:

What has it been like taking part in the intervention?

How has it changed your practice if at all?

What perceived benefits, downsides, and unintended consequences both positive and negative?

How has it affected the roles of the nurse and doctor and team working in general? How has it affected the management of LTCs? (*Goal setting?)*

How do you think this intervention and your role in it supports patient-centred care, if at all?

How do you think patients have responded? Who do you think has benefitted most/least?

What difficulties have there been in delivering the intervention? *(How easy was it to organise their care in this way?)*

What helped to deliver the intervention? *(whole system change or pockets?)*

How adequate was the preparation by, and support from, the research team?

How you were able to integrate the template into your consultation or not i.e. did you use it? We realise it is not ideal for everyone and would like to know how it could be improved?

Are there any elements of the intervention that are particularly useful or need changing?

Identifying concerns

Depression screening
Goal negotiation
Care plans
Length of appointment
Pharmacy review
Continuity of care

2. Have your views on the intervention changed in any way from when it was first introduced?

3. Specific questions to follow up on early interview or on observed consultation

4. Maintenance:

What would encourage you to keep this system of care for multi-morbid patients?

What will you do now? Are there any elements you might take forward? If so why and if not why not? *(Distinguish between concept not being enough of a priority (if so why not?) and whether or not this is the right way to do it)*

Does anything need to change? What would make it easier to implement? What would you do differently?

How have local circumstances affected what you did? Has that changed during the study?

5. Is there anything else you would like to say?

Topic guide for patients – post intervention .

Focus group or individual interviews

Can you comment on the care you receive from your GP practice in general and for your long-term conditions in particular?

What is most important to you about the way your care is provided?

Is there anything that you would like to change/improve? If so how?

What do you think of the 3D system?

What, if anything, is different about your care?

Has it had any effect on your health?

Have you had any care or intervention that you don't think you would have had without 3D?

Would you like to see the 3D system continuing?

If it was not all continued what would be the most important parts to continue?

Consultation observation guide

Patient identification code:

Clinician: **GP practice:**

Conditions reviewed:

3D review part 1 or part 2:

Length of consultation: scheduled: actual:

For each consultation note:

- General appearance and demeanour of clinician and patient
- Physical set-up of the room e.g. location of computer in relation to clinician/patient (diagram)
- How the consultation is opened
- Whether/how the clinician talks about 3D and how it may impact organisation of the patient's care
- Actions taken by clinician since last appointment and the responses of patient/carer
- Actions taken by the patient since last appointment and the responses of the clinician
- How the 3D template is used and talked about by the clinician during the consultation
    - *e.g. does the clinician refer to it or use it as justification for certain questions? Does it impact clinician/patient verbal/non-verbal communication? Are there any technical problems with use of the template?*
- How the patient/carer appears to respond to use of the template during the consultation
    - *e.g. any comments made by the patient or questions asked about use of template. Does the patient welcome the provision of written agenda/care plan or not?*
- Whether/how the clinicians seeks to elicit the patient's concerns and priorities
- Was everything covered i.e. was it truly holistic and was everything that might affect health and wellbeing considered?
- What the clinician tells the patient about their condition(s) and the responses of patient/carer
- Information and knowledge exchange: Were appropriate questions asked by both patient and doctor and were the answers adequate and did the doctor check understanding?
- How medication adherence is discussed and medications reviewed
- How depression is discussed
- How treatment/care plans are talked about and negotiated – are goals set?

- Was there evidence at the end of agreement as to what needed to be addressed and how that would be done?
- Nature of the clinician/patient relationship and decision making during the consultation
    - *e.g. examples of patient-centredness, who is managing the consultation agenda, involvement of patient/carer in care and treatment planning, clinician respect for patient's values/preferences, checking understanding*
- Interaction (verbal and non-verbal) between clinician and patient/carer during the consultation
    - *e.g. how questions are asked, responses to questions, verbal/non-verbal cues, clinician empathy, eye contact*
- Was it genuinely open or were closed questions asked that limited the scope?
- What was the last thing the patient said?
- How the consultation is closed, including discussion of plans for the next review
- Any other relevant issues
