# Supplementary material for: Can implementation failure or intervention failure explain the result of the 3D multimorbidity trial in general practice: mixed-methods process evaluation
Source: BMJ Open. 2019 Nov 6;9(11):e031438. doi: 10.1136/bmjopen-2019-031438 (PMC6858134; doi:10.1136/bmjopen-2019-031438)
Supplement: Supplementary data [file bmjopen-2019-031438supp003.pdf]

Appendix 3

Consolidated criteria for reporting qualitative studies (COREQ): 32-item checklist

1.	Interviewer/facilitator	The interviews, focus groups and observations were conducted by Cindy Mann, with the exception of 5 observations and one interview that were carried out by Polly Duncan.
2.	Credentials	Cindy Mann had an MSc and previous qualitative research experience at the time of the study. Polly Duncan is an academic GP and was gaining qualitative research experience at that time.
3.	Occupation	CM was a senior research associate, Polly Duncan was a GP with an academic training fellowship
4.	Gender	Both female
5.	Experience and Training	CM has training and over 5 years experience in qualitative research and research methods. Experience in various environments (primary care and secondary care) as a researcher, research nurse and clinical nurse and experience as a counsellor and group facilitator. PD is a qualified GP with additional academic experience of research.
6.	Relationship established	Prior to study commencement, the interviewer and the participants had no previous contacts. Rapport was built before interview, focus groups or observations by answering questions from participants and taking informed consent.
7.	Participant knowledge of the interviewer	The participants did not have prior knowledge of the interviewer before the study. When participants were recruited, they were provided with an information leaflet about the study and purpose of the interview/focus group/observation which was repeated prior to data collection beginning. Information about the researcher was not provided other than her role in the research team.
8.	Interviewer characteristics	The principle qualitative researcher (CM) is a white, university-educated British woman with nursing, counselling and research qualifications. Qualitative research is always influenced by the perspective of the researcher, and in this case the nursing perspective and

	primary care clinical experience may have fed into the way some clinical participants were interviewed.
9. Methodological orientation and Theory	The key methodological framework used was a framework for process evaluation for cluster randomised trials and the MRC guidance for the process evaluation for complex interventions framework. Mixed methods were used, and thematic analysis was used for the qualitative data.
10. Sample	Intervention practices taking part in the 3D trial were purposively sampled for the process evaluation based on their characteristics. Individual staff members and clinicians of those practices that agreed to take part in the process evaluation were separately invited to take part in the process evaluation, based on their roles. Patient participants were sampled based on their responses to a baseline questionnaire.
11. Method of approach	Patient participants were approached by invitation letter including information sheet and staff and clinicians by email with invitation letter and information attached. In both cases follow up contact was made to discuss possible participation and to arrange the details.
12. Sample Size	The total number of interviews with staff, including informal debriefs after 3D reviews, was 32 (18 GPs, 20 nurses and 9 administrator interviews). Some individuals were interviewed twice so the actual number of those interviewed was 11 GPs, 14 nurses, 7 administrators and 38 patients (including the 22 patients who attended a focus group). 28 intervention review observations were carried out.
13. Non-participation	Some patients refused interviews or focus group and 1 nurse refused review observation
14. Setting of Data Collection	Interviews were conducted in GP practices, patients' homes or, in the case of focus groups, local halls, depending on convenience and patient preference. Observations were all carried out at the GP practice.
15. Presence of non-participants	Patients' carers were sometimes present at review observations, interviews or focus groups but all of them also provided consent. The researcher was present in a non-participatory role at observations

16. Description of the sample	GPs, administrators, practice nurses and patients from 9 different GP practices
17. Interview guide	Interview guides, a focus group schedule and an observation guide were used to act as a checklist but without imposing a set structure
18. Repeat interviews	Repeat interviews were carried out with some nurses, GPs and administrators who were interviewed both at beginning and end of the trial
19. Audio-/visual recording	We used audio recording to collect all data.
20. Field notes	Field notes were made during the observations to note participant expression, or other non-verbal cues and in all instances of data collection to describe the ambience of the GP practice and reception and aspects of the environment and interaction.
21. Duration	Pre-arranged interviews lasted 15-50 minutes and follow-up interviews lasted 5-24 minutes. Focus groups lasted an hour. Review observations lasted between 20 and 60 minutes.
22. Data Saturation	The concept of information power was used, rather than data saturation, since it is more in keeping with the process evaluation focus.
23. Transcripts returned	Transcripts were not returned to participants for comment or correction.
24. Number of data coders	One (Cindy Mann), with double coding of a sub-sample by Alison Shaw, Lesley Wye, Polly Duncan and 2 members of the Patient Public Involvement group
25. Description of the coding tree	Not included in this manuscript because the purpose of this paper is not primarily to report the findings of a qualitative piece of research
26. Derivation of themes	Themes in the qualitative data were a priori based on intervention components, supplemented by themes identified in the data
27. Software	NVivo v11
28. Participant checking	No. Transcripts were not returned to the participants for checking.

29. Quotations presented	Yes, participant quotations are presented to illustrate the themes.
30. Data and findings consistent	Yes.
31. Clarity of major themes	Major themes are based around intervention components as the purpose of the paper is to assess implementation fidelity
32. Clarity of minor themes	Not applicable
