# Supplementary material for: Can implementation failure or intervention failure explain the result of the 3D multimorbidity trial in general practice: mixed-methods process evaluation
Source: BMJ Open. 2019 Nov 6;9(11):e031438. doi: 10.1136/bmjopen-2019-031438 (PMC6858134; doi:10.1136/bmjopen-2019-031438)
Supplement: Supplementary data [file bmjopen-2019-031438supp004.pdf]

Appendix 4: Electronic monitoring of review component delivery

Practice	Penn	Priestman	Sharples	McReady	Harvey	Blackwell	Guppy	Lovell	Tothill	Beddoes	Dunbar	Plimsoll[1]	Carpenter	Davy	Cabot	Martineau	ALL
**3d agenda printed**	97%	92%	100%	89%	**97%**	81%	95%	**98%**	98%	**100%**	100%	70%	97%	**58%**	100%	92%	**96%**
**3d health plan printed**	77%	81%	97%	91%	**62%**	31%	23%	**100%**	80%	**98%**	85%	39%	85%	**80%**	98%	67%	**83%**
**adherence meds**	95%	61%	94%	96%	**65%**	92%	63%	**100%**	39%	**67%**	62%	44%	54%	**50%**	93%	64%	**71%**
**EQ5D pain**	47%	97%	100%	71%	**100%**	96%	65%	**52%**	100%	**98%**	100%	5%	100%	**100%**	100%	95%	**83%**
**GP first goal noted**	100%	97%	100%	100%	**76%**	96%	100%	**100%**	102%	**98%**	102%	44%	100%	**95%**	93%	97%	**94%**
**Most important problem on nurse view**	100%	97%	100%	100%	**100%**	96%	100%	**100%**	100%	**100%**	100%	100%	100%	**100%**	100%	97%	**99%**
**Pharmacist comment**	84%	100%	95%	82%	**105%**	78%	83%	**100%**	107%	**106%**	100%	38%	78%	**43%**	100%	102%	**88%**
**Pharmacist comments noted?**		56%	53%		**47%**	77%			68%	**69%**	92%		56%	**80%**	95%	64%	**69%**
**PHQ9 done**	97%	97%	100%	91%	**91%**	96%	98%	**100%**	98%	**98%**	100%	100%	97%	**94%**	100%	103%	**98%**
**what GP can do about main problem**	92%	89%	100%	98%	**71%**	73%	98%	**76%**	100%	**89%**	102%	33%	87%	**80%**	90%	72%	**84%**
**what patient can do about main problem noted**	77%	92%	86%	96%	**76%**	73%	100%	**91%**	100%	**93%**	100%	39%	97%	**85%**	90%	78%	**86%**
**3D participants Pharmacist comment**	84%	100%	95%	82%	**105%**	78%	83%	**100%**	107%	**106%**	100%	38%		**43%**	100%	102%	**88%**

Key: Range of fidelity from red (worst) to green (best)
Grey-shaded column headers indicate case study practices
Practice ID: 60 = Harvey; 46 = Lovell; 26 = Beddoes; 69 = Davy
Some values are greater than 100% because percentages were calculated based on the number of participants remaining in the trial at the end
[1]*This practice stopped delivering the intervention and withdrew from the process evaluation*
