# Supplementary material for: Can implementation failure or intervention failure explain the result of the 3D multimorbidity trial in general practice: mixed-methods process evaluation
Source: BMJ Open. 2019 Nov 6;9(11):e031438. doi: 10.1136/bmjopen-2019-031438 (PMC6858134; doi:10.1136/bmjopen-2019-031438)
Supplement: Author's manuscript [file bmjopen-2019-031438.draft_revisions.pdf]

BMJ Open is committed to open peer review. As part of this commitment we make the peer review history of every article we publish publicly available.

When an article is published we post the peer reviewers' comments and the authors' responses online. We also post the versions of the paper that were used during peer review. These are the versions that the peer review comments apply to.

The versions of the paper that follow are the versions that were submitted during the peer review process. They are not the versions of record or the final published versions. They should not be cited or distributed as the published version of this manuscript.

BMJ Open is an open access journal and the full, final, typeset and author-corrected version of record of the manuscript is available on our site with no access controls, subscription charges or pay-per-view fees (http://bmjopen.bmj.com).

If you have any questions on BMJ Open's open peer review process please email info.bmjopen@bmj.com

BMJ Open

Distinguishing implementation failure from intervention failure: process evaluation of the 3D multimorbidity trial

Journal:	*BMJ Open*
Manuscript ID	bmjopen-2019-031438
Article Type:	Research
Date Submitted by the Author:	03-May-2019

Complete List of Authors:	Mann, Cindy; University of Bristol School of Social and Community Medicine, Academic Primary Care<br>Shaw, Ali; University of Bristol, School of Social and Community Medicine<br>Guthrie, Bruce; University of Edinburgh College of Medicine and Veterinary Medicine,<br>Wye, Lesley; University of Bristol, Academic Unit of Primary Health<br>Man, Mei-See; University of Bristol School of Social and Community Medicine,<br>Chaplin, Katherine ; University of Bristol Centre for Academic Primary Care, Bristol Medical School<br>Salisbury, Chris; University of Bristol, Academic Unit of Primary Health Care

Keywords:	Process evaluation, Multimorbidity, Implementation fidelity, PRIMARY CARE, Null trial, Patient-centred

SCHOLARONE™
Manuscripts

Distinguishing implementation failure from intervention failure: process evaluation of the 3D multimorbidity trial

Cindy Mann, Alison Shaw, Bruce Guthrie, Lesley Wye, Mei-See Man, Katherine Chaplin, Chris Salisbury

Cindy Mann[1], cindy.mann@bristol.ac.uk **Corresponding author**

Alison Shaw[1], ali.heawood@bristol.ac.uk

Bruce Guthrie[2], bruce.guthrie@ed.ac.uk

Lesley Wye[1], lesley.wye@bristol.ac.uk

Mei-See Man[1], mei-see.man@bristol.ac.uk

Katherine Chaplin[1], k.chaplin@bristol.ac.uk

Chris Salisbury[1], c.salisbury@bristol.ac.uk

[1]Centre for Academic Primary Care, NIHR School for Primary Care Research, Population Health Sciences, Bristol Medical School, University of Bristol, 39 Whatley Road, Bristol BS8 2PS, UK

[2] Usher Institute of Population Health Sciences and Informatics, College of Medicine and Veterinary Medicine, University of Edinburgh, Old Medical School, Teviot Place, Edinburgh, EH8 9AG, UK

For peer review only - http://bmjopen.bmj.com/site/about/guidelines.xhtml

Abstract

Objectives, design and setting

A process evaluation was conducted alongside a cluster-randomised trial (The 3D Study), involving 1546 participants with multimorbidity in 33 UK general practices. The trial intervention enacted recommended care for people with multimorbidity including continuity of care and comprehensive biennial patient reviews supported by a purpose-designed electronic template. The mixed-methods process evaluation aimed to inform future implementation by examining implementation variation and fidelity.

Methods

Qualitative data (interviews, focus groups and review observations) were obtained from 19 clinicians, 7 administrators and 38 patients, analysed thematically and integrated with quantitative data about implementation fidelity collected via the electronic template from all implementation practices. Analysis was blind to trial outcomes (null for quality of life and health, positive for patient-centredness) and examined context, intervention adoption, reach and maintenance, and delivery of reviews to patients.

Results

Staff loss, practice size and different administrative strategies influenced implementation fidelity. Practices with whole administrative team involvement and good alignment between the intervention and usual care generally implemented better. Fewer reviews than intended were delivered (49% of patients receiving both intended reviews, 30% partially reviewed). In completed reviews >90% of intended components were delivered but review observations and interviews with patients and clinicians found variation in style of component delivery, from ‘tick-box’ to patient-centred approaches. Implementation barriers included lack of skills training to implement patient-centred care planning, but patients reported increased patient-centredness due to comprehensive reviews, extra time and being asked about their health concerns.

For peer review only - http://bmjopen.bmj.com/site/about/guidelines.xhtml

Conclusions

Implementation failure contributed to lack of impact of the 3D intervention on the trial primary outcome (quality of life), but modifiable elements of intervention design were partially responsible. When a decisive distinction between implementation failure and intervention failure cannot be made, identifying potentially modifiable reasons for sub-optimal implementation can inform a re-designed intervention for further evaluation and/or wider implementation.

Trial registration number

ISRCTN06180958 registered 18.2.2014

Key words

Process evaluation, implementation fidelity, multimorbidity, primary care, cluster-randomised trial, null trial

Strengths and limitations of this study:

- In the largest randomised controlled trial of a recommended patient-centred model of care for people with multimorbidity, we conducted a comprehensive process evaluation to examine implementation fidelity in case of a null result and to inform future implementation.
- We used mixed methods to evaluate multiple aspects of implementation and a wide range of factors that might influence implementation.
- Although distinguishing between implementation failure and intervention failure is recommended in null trials to avoid needlessly discarding a promising intervention, the distinction is difficult to apply when aspects of intervention design contribute to implementation deficiencies.

For peer review only - http://bmjopen.bmj.com/site/about/guidelines.xhtml

- By investigating reasons for implementation deficiencies, and distinguishing between potentially modifiable and non-modifiable reasons, we have instead provided information that is potentially more valuable than dichotomising between implementation failure and intervention failure for informing decisions about wider implementation, or the need for further research.

Introduction

For peer review only

The increasing prevalence of multimorbidity, driven by aging populations across the world, is a major challenge to health services. Reflecting an absence of evidence, the 2016 National Institute of Health and Care Excellence Multimorbidity clinical guideline recommended more research on how best to organise primary care to address these challenges [1]. There is broad consensus about how primary care for people with multimorbidity should be organised, [1-3] but little evidence about the effectiveness of recommended strategies. In the largest trial to date of an intervention based on this consensus, the 3D study evaluated a patient-centred approach that provided regular holistic reviews (3D reviews) in primary care (General Practices in the UK) with a focus on addressing quality of life, mental as well as physical health, and polypharmacy. The hypothesis was that this would improve patient-centred care, reduce treatment burden and illness burden and improve quality of life (the trial primary outcome) [4].

Process evaluation of trials evaluating complex interventions can inform decisions about the wider implementation and applicability of interventions shown to be effective in trials. A comprehensive process evaluation can help interpret trial results and inform real-world implementation [5, 6]. By examining implementation fidelity, process evaluation can also provide explanations for why interventions are not effective [7]. This may be because of *intervention failure* (the intervention was delivered as intended but did not improve outcomes, so should not be implemented) and/or *implementation failure* (the intervention was inadequately implemented and so might need

For peer review only - http://bmjopen.bmj.com/site/about/guidelines.xhtml

additional research to further examine effectiveness) [8]. However, distinguishing implementation and intervention failure is often not straightforward [9, 10].

We have previously reported baseline data from the 3D Study [11], main trial findings [12, 13] and analysis of the patient-centredness of the 3D review [14]. At baseline, many practices had already combined multiple long-term condition reviews into one appointment but other recommended care [1, 2] was less evident. For example, only 10% of patients were aware of receiving a care plan and 35% were rarely or never asked what was important to them in managing their health [11]. The main trial results showed no effect from the 3D intervention on the primary outcome of health-related quality of life (HR-QOL) or other related secondary outcomes such as wellbeing and treatment burden, but a consistent beneficial effect on patients' experience of care as more person-centred [12]. Analysis of observational and interview data about intervention delivery indicated that the main reasons for the perceived increase in patient-centredness were that when patients attended for an intervention review, they were first asked about their most important health concerns and then given a longer, comprehensive review encompassing all health issues [14]. The aim of this paper is to examine whether the observed lack of effect on the primary outcome in the 3D trial was due to implementation or intervention failure, with a view to interpreting trial findings, enhancing impact and informing future intervention implementation [15].

Methods

Setting - The 3D study

The intervention, trial evaluation and process evaluation are described briefly here, having been reported in detail elsewhere [4, 12, 13, 16]. The core components of the intervention included offering greater continuity of care and six-monthly, two-part patient-centred, comprehensive health reviews, conducted by a named nurse and GP and underpinned by a purpose-designed electronic template (Figure 1). A pharmacist also completed an electronic medication review. Practices were expected to deliver two complete reviews to every patient during the trial, including all review

For peer review only - http://bmjopen.bmj.com/site/about/guidelines.xhtml

components. However, practices could decide the detail of how they would provide the reviews, enhance continuity of care and reduce the number of review appointments. Administrators and clinicians nominated by the practices received two short (2-3 hours) training sessions from the trial team on the intervention's rationale and the use of the computer template. Additional file 1 shows the TIDieR checklist [17] for the intervention design. Figure 1 details the work that administrative staff, clinicians and pharmacists were expected to do to deliver the intervention. Sixteen general practices received the intervention compared to 17 control practices, with 1546 participating patients [4]. However, because of staffing crises, one intervention practice stopped delivering the intervention and withdrew from the process evaluation.

Patient and Public involvement

A patient public involvement group was set up during development of the trial intervention pre-funding to ensure that it met the perceived needs of people with multimorbidity. The group was actively involved throughout the trial in multiple ways, as reported by Mann et al. [18].

Process evaluation design

The design [16] was based on a process evaluation framework for cluster randomised trials [19], and informed by UK Medical Research Council guidance for process evaluation of complex interventions [10]. We based the process evaluation on a logic map describing the intervention design and used the logic map to inform assessment of implementation fidelity (the extent to which practices implemented the intervention as the researchers intended) [16]. The assessment covered adoption of the 3D intervention (implementation of the organisational components of the intervention); delivery of 3D reviews to patients; maintenance (whether delivery is sustained over time) and reach (the number of participants who receive the intervention) (Figure 2), and the important influence of context on implementation fidelity, maintenance and reach [20-23].

For peer review only - http://bmjopen.bmj.com/site/about/guidelines.xhtml

Data collection

Qualitative data collection in selected practices

We selected nine out of the 16 intervention practices for qualitative data collection at different stages (Table 1). Four practices were initially purposefully sampled during early stages of the trial, using baseline data and observation of practice team training, for detailed qualitative investigation of all aspects of implementation. Five were responsively sampled at later stages for focused observation of clinicians' style of delivery of 3D reviews and to examine variations in models of delivery that emerged during the trial. Initial sampling of the four practices reflected our assumptions that (a) larger practices may have lower continuity of care and a lower proportion of clinicians taking part in 3D which may influence implementation; and (b) practices whose care for patients with multimorbidity already reflected aspects of the 3D approach may adopt 3D more readily. The five responsively sampled practices included one practice where a research nurse was responsible for arranging 3D reviews and delivering the first part of each review, and another where a nurse practitioner delivered both parts of the 3D reviews to all patients.

All intervention practices were given pseudonyms to preserve anonymity. Data collected included: interviews with practice staff; non-participant observation of 3D reviews with follow-up interviews with clinicians and patients; and focus groups and interviews with patients (Table 1), all of which were audio-recorded.

Interviews with practice staff: At baseline, interviews in the four initially-sampled practices with the 3D lead GP, the lead nurse and the key administrator explored usual care, initial reactions to the intervention and implementation arrangements. Interviews at the end of the trial in the same four practices, and in the practice where a nurse practitioner delivered all reviews, explored experience of delivering the intervention and maintenance. Interviews lasted 15-50 minutes and some individuals were interviewed on more than one occasion during the trial.

For peer review only - http://bmjopen.bmj.com/site/about/guidelines.xhtml

Observation of 3D reviews with follow-up interviews: Twenty-eight 3D reviews were observed and recorded in the four initially-selected practices and four responsively sampled practices, and observation notes made. Where possible, brief follow-on interviews with the clinician and/or patient whose review had been observed were completed on the same day.

Focus groups and interviews with patients: In the four initially-sampled practices, patients varying in health status and satisfaction with care according to baseline questionnaire data were invited to focus groups or individual interview towards the end of the trial, to explore their experience of receiving the intervention. One focus group per practice took place, lasting about one hour. Patients preferring individual interviews were interviewed for 20-50 minutes in a convenient location, usually their own home.

Additional file 2 shows the COREQ checklist [24] for qualitative methodology.

Quantitative data collected from all intervention practices

Data about 3D review completion were extracted each month from the routine electronic medical records to evaluate intervention reach, delivery and maintenance [4, 16]. The data included dates of reviews, who had completed the review, and whether core elements were recorded as delivered in the 3D review template. In the first part of the review delivered by a nurse, data included completion of patients' main concerns, pain levels, depression screening, and the creation and printing of a patient agenda. The template also recorded the pharmacist's completion of a medication review, their recommendations and whether these had been noted by the GP. In the second part of the review delivered by a GP (except in one practice), recorded data included medication adherence and description of at least one main problem in the health plan, together with patient and GP actions to address the problem. Finally, the software recorded whether an agreed health plan had been printed.

For peer review only - http://bmjopen.bmj.com/site/about/guidelines.xhtml

Survey data collected in all intervention practices

Researchers in each trial area completed a purpose-designed administrative survey about the way 3D reviews were organised in all intervention practices. The survey included the proportion of the administrative team involved in 3D, how patients were identified and contacted, and whether practices facilitated 3D patients seeing their named GP at appointments other than 3D reviews.

Data analysis

All audio-recordings of qualitative data were professionally transcribed, then the transcript was checked against the recording, anonymised and annotated with observation notes. The data were used to write detailed qualitative description [25] of context and adoption of the intervention in the four practices initially sampled for detailed examination, and for cross-case thematic analysis [26] of recurring issues relevant to intervention delivery and maintenance in all nine selected practices. The data were analysed in parallel with data collection, so that emerging issues were incorporated into future data collection. For the thematic analysis, NVivo v.11 software (QSR International) was used to facilitate both deductive coding derived from intervention components and inductive coding arising from the data [26], allowing the identification of both anticipated themes (e.g. those relating to the key components of the intervention) and emergent themes across sampled practices. Qualitative analysis was led by CM with input from AS, LW and BG, who commented on the developing coding framework, double-coded a sample of transcripts and agreed the final themes. Additionally, to further enhance trustworthiness and credibility of findings, two members of the Patient and Public Involvement group each coded four transcripts to check interpretation of the data from the patient perspective. Quantitative data were analysed descriptively by CM and KC and integrated with qualitative data.

All process evaluation data collection and analyses were done blind to the trial outcome, so that interpretation would not be influenced by knowing the results of the primary outcome.

For peer review only

For peer review only - http://bmjopen.bmj.com/site/about/guidelines.xhtml

Results

The results examine 1) Adoption of the intervention by practices, 2) Reach and maintenance, and 3) Delivery of reviews to patients. In quotes, staff and patients are identified by practice pseudonym, role and a number.

Adoption – organisational components

The two core components of organisational adoption were continuity of care and arranging the two-part 3D reviews.

Continuity of care

Practices were asked to allocate a named GP to 3D patients for their reviews and for any appointment between reviews. Continuity of care was evaluated as a secondary outcome for the trial and, measured using the Continuity of Care index [27], increased slightly in the intervention arm [12]. However, some patients experienced reduced continuity because their GP left during the trial. Others were allocated a different GP for the intervention, either to share work-load or because their usual GP was not participating in 3D. These patients often continued to see their usual GP for appointments other than reviews.

> *[My usual GP] had to get changed. There's three doctors in our practice and they were doing I think 12 patients, so it was split between three doctors. So I had to go with [GP2]. (Focus group Lovell Patient 8)*

The four initially-sampled practices (Beddoes, Davy, Harvey and Lovell) provided insight into contextual influences. Harvey already had a "personal list" system with high continuity, but during the trial this was disrupted when several GPs left the practice. Beddoes supported 3D participants to see their allocated GP between reviews. At Davy, continuity was poorly implemented due to staff loss and because receptionists were unaware of 3D. Lovell continued with their usual system, which they felt delivered adequate continuity of care.

For peer review only - http://bmjopen.bmj.com/site/about/guidelines.xhtml

For peer review only

218 *Most people see the doctor they want to see, so I think from a continuity point of view*
219 *we know our patients very well and we've all been here a long time. [Group interview*
220 *Lovell GP1]*

221 *Arranging reviews*

222 Administrative survey data from 15 intervention practices showed variation in the way practices
223 arranged reviews (Table 2). Ten practices involved the whole administrative team, but in four, one or
224 two administrators arranged 3D reviews in isolation. Reach was lowest in these four practices. In the
225 remaining practice (Cabot), a dedicated research nurse arranged all the reviews, bypassing the
226 administrative team. Notably, some 3D patients received the 3D reviews in addition to, rather than
227 instead of their usual individual condition reviews, as intended.

228 *I think there became a problem where patients were being invited in for their 3D and*
229 *then a couple of months later, they'd get invited in for their diabetes and their asthma*
230 *because one person up there wasn't talking to the other one. [Interview Blackwell Nurse*
231 *1]*

232 At Lovell and Harvey, existing arrangements for long-term condition reviews (one of the sampling
233 criteria) underpinned the 3D review arrangements, reducing confusion. At Davy, the two
234 administrators involved had to set up a different system for 3D patients. Being a large practice in
235 which the rest of the administrative team were unaware of 3D requirements, difficulties arose when
236 patients needed to re-arrange the appointment. At Beddoes, clinical and administrative staff
237 decided collectively how they would implement the administrative aspects of 3D, but it differed
238 from usual arrangements.

239 *We'd had a team meeting after the training with the senior nurse and the GPs to decide*
240 *what was the best way forward and then I met with the admin team to say, "What*
241 *would you like to see on your screen so that you know they're part of the 3D study and*
242 *so that you know about the appointments?" (Interview Beddoes practice manager)*

For peer review only - http://bmjopen.bmj.com/site/about/guidelines.xhtml

Overall, adoption was inconsistent, affected by practices' choices in respect of continuity and arrangements for reviews Duplication of reviews in some practices suggests difficulty in testing effectiveness of an intervention in a research situation that involves a short-term alteration to accustomed methods of providing care that affects only a sub-set of patients.

Reach and maintenance

Table 3 shows mean reach in all intervention practices. We defined intervention reach in terms of receipt of planned 3D reviews by participating patients. Reach varied between practices from 38% and 94% (median 66%) of all recruited patients in a practice receiving both the nurse and GP appointments in first round reviews, and between 0% and 93% (median 47%) in second round reviews. Initial implementation of the intervention was therefore not well-maintained.

In the four initially-sampled practices, the qualitative data revealed contextual factors reducing the time window for delivering reviews. Lovell started delivering 3D reviews straight after training and had the highest reach of any practice in the intervention arm. The other three practices delayed starting, Davy because of the sudden loss of three of their long-term condition nurses and two GPs, Harvey because they were changing their system for sending letters re-calling patients for long-term reviews, and Beddoes because of staff sickness. Once started, Davy administrators struggled to organise reviews, hampered by ongoing sickness in the nursing team, and only managed to schedule 25% of the reviews required. The greatest challenge was accommodating paired reviews within over-stretched appointment schedules.

> *And I think because you're trying to tally it up with the doctor and the nurse, trying to find the time with the nurse if they've got more than one problem … and again they're not full time; they work part time. [Interview Davy Administrator 1]*

Difficulties with arranging appointments reinforced practices' initial fears that the time demand and workload of implementing the 3D intervention would be too great. One suggestion made by GPs was

For peer review only - http://bmjopen.bmj.com/site/about/guidelines.xhtml

267 that patients could be selected using more stringent criteria to reduce the overall number and
268 maximise the chance of benefit. Another suggestion, from nurses, GPs and patients, was that the
269 reviews need not involve the GP every time and/or could be shorter. Some comments suggested a
270 lack of perceived value of the second-round reviews and that a second-round review with the nurse
271 alone would be more time-efficient.

272 *I know they need to be reviewed but do they need to be reviewed by nurse and GP?*
273 *… because if we saw them for review and they were happy. Do they honestly need to*
274 *see the GP to say "Are you still happy, like from last week"? [Interview Guppy Nurse 1]*

275 Practices may therefore have been less motivated to arrange second reviews, and one practice
276 reported that fewer patients responded to the invitation to attend them.

277 *As a practice we've actually struggled to get them in for their second ones … we've*
278 *written to them all twice – probably 30% of them haven't booked in and so we have had*
279 *a bigger DNA rate for the second ones than the first ones [Interview Beddoes GP1]*

280 Overall, reach and maintenance were lower than intended, indicating a degree of implementation
281 failure. Attention to context showed this was mainly a result of unanticipated events (e.g. staff loss
282 or sickness) affecting practice capacity. However, aspects of intervention design (e.g. the inclusion of
283 two reviews in one year with both nurse and GP each time) may also have impacted reach and
284 maintenance.

285 **Delivery of 3D review components**

286 In 3D reviews that took place, each of the intervention components (see Figure 1) detected by the
287 electronic search were completed in at least 92% of the delivered reviews, except medication
288 adherence which was completed in 84% and printing the health plan in 77% (Table 4 and Additional
289 file 3). The qualitative data provided insight into reasons for less consistently recorded components
290 but also found evidence of significant variation in the manner of delivery suggesting that the high

For peer review only - http://bmjopen.bmj.com/site/about/guidelines.xhtml

recorded component completion concealed some tick-box compliance. Variation in the patient-centredness of review component delivery has been reported in more detail in a previous paper [14]; here we focus primarily on implementation fidelity.

Eliciting and documenting the patient's concerns (most important problem noted)

The most consistently delivered component (99% completion) (Table 4), was asking patients about the health problems important to them. Nurses often invited disclosure of all health concerns, large or small.

> *She said to me, 'Is there anything you want to discuss with me at all, anything?' [Focus group Beddoes Patient 4]*

Some GPs and nurses commented on the value and novelty of asking about all patients' health concerns at the start of the consultation [14] but others were conscious of their clinical responsibility for managing the long-term conditions. Therefore, they preferred to separate the long-term conditions from health concerns they viewed as more trivial, or disabilities not amenable to change.

> *They want to discuss … the things that are happening to them at that particular moment … they've got a bad cold, or the cat's died or something else and they don't want to talk about their diabetes or their COPD. [Interview Beddoes GP3]*

There was also observed variation in how patient's concerns were elicited, recorded in the agenda and addressed in the health plan. The printed agenda was intended to reflect the patient's perception of health problems (as well as clinical concerns), but nurses were often observed to reframe patients' problems into more medical terms. For example, one patient said: '*I can't take these naproxen now because … they've upset my stomach'* and the nurse recorded 'gastric problems'. This medicalisation of problems may have contributed to some patients' perception that the agenda was simply a means for the nurse to communicate their findings to the GP, rather than an agenda that the patient owned.

For peer review only

For peer review only - http://bmjopen.bmj.com/site/about/guidelines.xhtml

> *They just went through everything, all the problems, the nurse did and just wrote this report out for [GP2]. [Focus group Beddoes Patient 11]*

Quality of life and depression screening

Although completion was high, observation revealed that components that had a range of set answers were sometimes delivered in a 'tick-box' way that did not invite dialogue. This most commonly happened with template questions about quality of life and depression screening. It usually occurred when the nurse anticipated no problems being revealed but in interview some nurses also said that they lacked confidence in talking to patients about mental health.

Printing patient agenda

The patient agenda was printed in the vast majority of cases (93%) (Table 4) but problems with printing were occasionally observed and one nurse said she asked patients if they wanted it and that they declined.

> *Would you like a copy? And they're like, it's fine…Nobody has wanted a copy. [Interview Davy Nurse 1]*

Medication adherence

The completion rate of this component was lower at 84% but the qualitative data did not reveal why, other than some GPs' preference to complete the template after the review, which may have meant they forgot to ask about it. On the contrary, there was evidence of some support for this component among GPs.

> *I do think the thing about tablets that patients take and which ones they don't like, if any, is useful. [Interview Lovell GP1]*

For peer review only

Collaboratively agreeing a plan

Health plans were intended as collaborative agreements between patient and GP, recording identified problems and specific actions for patient and GP to address each recorded problem. The patient and GP actions were well completed (93% and 92% respectively for the first problem) but the health plan was printed less frequently (77%) (Table 4). This may reflect GPs apparent dislike of the health plan and a perceived lack of value, as well as technical difficulties printing the plan. Interview data included reservations about the formulation of the health plan, which may have made GPs reluctant to give them to patients.

> *I felt it was almost that you were actually chiding them in some ways, to say, 'You should do this, should do that. … It's almost like when we were at primary school, taking home your homework tasks and goals for the week' [Group interview Lovell GP3]*

During observations, a collaborative dialogue based on patients' chosen goals was seldom generated, and most plans were based on actions suggested by the GP. Some GPs commented that patients had not given prior thought to what they wished to address and that sometimes it was difficult to identify problems to include in the plan.

> *That's where I think perhaps them thinking in advance about their goal setting would help aid the conversation because often they say "No, no there's nothing I want to discuss" and you eventually tease out one or two things from them. [Interview Beddoes GP1]*

Some clinicians felt that the training provided by the trial team was insufficient to enhance skills required for agenda setting and especially collaborative action-planning.

> *I think some kind of communication training … would have been useful…there was a little bit about goal setting and confidence skills but there was no real practical element to it so in some ways you're testing what we already do but in a different context [Interview Lovell GP1]*

For peer review only - http://bmjopen.bmj.com/site/about/guidelines.xhtml

Others would have liked some training follow-up to check if they were delivering the intervention as intended, and additional training prior to the second round of reviews to ensure they were '*doing it right*'.

In conclusion, although the quantitative data indicated that the intervention components were delivered for a high proportion of patients receiving reviews, the qualitative data showed that delivery style varied in ways that could sometimes compromise their function. Some components, such as creating the health plan, could have benefitted from more training.

Discussion

Summary of findings

The process evaluation identified that implementation was somewhat deficient in adoption (arranging the requisite number of 3D reviews, ensuring continuity of care, reducing the overall number of reviews) and aspects of delivery (creating health plans), but most delivered reviews included all components. Reasons for incomplete implementation included unexpected pressure on resources, implementation choices made by practices (including not involving the entire administrative team), and insufficient training for using patient-centred approaches. During delivery of reviews to patients, using the template was the key to maintaining 'fidelity of form', but variation in the patient-centredness of delivery sometimes undermined 'fidelity of function' [28]. The overall prediction made by the process evaluation team while blind to the trial results was that the intervention would have improved patient experience in patients who received 3D reviews, but not changed health-related quality of life (the findings were presented and this prediction made at the Trial Steering Committee meeting immediately before unblinding). The trial results confirmed this prediction [12], which increases our confidence in the process evaluation findings.

For peer review only - http://bmjopen.bmj.com/site/about/guidelines.xhtml

Strengths and weaknesses

Strengths include pre-designing the process evaluation based on a published framework for process evaluation of cluster-randomised trials [10, 16, 19] covering all trial stages, and maintaining responsiveness to emerging information. This maximised the likelihood that all factors that might influence implementation fidelity, including context, were considered [7]. Data of varying and complementary types were collected from a wide range of sources, both purposively sampled and cross-trial. The purposive sampling of practices mitigated the limitation that only a subset of practices and individuals involved in the trial were interviewed or observed, and we explored the full range of variation in implementation and reach (Table 2), including quantitative process data from all practices. In accordance with published guidance [10], the process evaluation analysis took place blind to the trial results.

Comparison to other literature

An aim of the 3D process evaluation was to examine implementation fidelity to distinguish between implementation failure and intervention failure in the event of a null result. This distinction matters because it is important to avoid discarding a potentially effective intervention that was poorly implemented [10, 29, 30]. Implementation difficulties and deficiencies are not infrequently identified in effectiveness evaluations of complex health care delivery interventions [31-34] but are not always elucidated [20, 35]. In this study we found evidence of a degree of implementation failure and, in addition to identifying poorly implemented components, we have considered reasons for poor implementation and whether they are modifiable. Non-modifiable reasons include unexpected events in individual practices, most commonly staff leaving and not being easily replaceable. Potentially modifiable reasons for adoption problems include the individual choices practices made about arranging reviews but implementation was also affected by the research trial context. Implementation in these circumstances is short-term, and only applies to a sub-set of patients, with the majority still receiving usual care, which increases the risk of confusion and

For peer review only

For peer review only - http://bmjopen.bmj.com/site/about/guidelines.xhtml

duplication. This circumstance influenced administrative choices made by practices, which in turn affected implementation.

The role of intervention design and set-up, including training provided by research teams to practices, is significant and modifiable. In common with other research teams, we experienced difficulty in establishing a new way of working [36, 37] but, care *did* change enough that patients reported statistically significant changes in their experience of care in the intended direction (e.g. having a greater sense of being consulted about their experience of health) and greater satisfaction with their care [12]. The evidence suggested that this was attributable to the design of the intervention reviews (longer, comprehensive, and asking first about the patient's concerns) [14] but there was also evidence that intervention design negatively affected implementation in some potentially modifiable ways. Implementation of health plans suffered from insufficient training and a lack of coherence between the health plan format and GP current practice, clearly suggesting that intervention design relating to both these aspects could be improved. Professional perceptions that some patients were unprepared to engage in health planning suggests that additional patient-targeted intervention components and/or better clinician training addressing attitudes and barriers to engaging in health-planning and supporting self-management [38] might facilitate collaboratively agreeing a plan of action. Many professionals did not see value for many patients in doing a second comprehensive review in the same year, which likely contributed to lower reach for second reviews, and suggests that more targeted follow-up might have been a better design than routine re-review for all.

Our overall judgement was that there was therefore evidence of both implementation failure and intervention failure, but that these were linked rather than truly distinct because in this case aspects of intervention design influenced implementation. Improvements in intervention design could be focused on incorporating skills practice in the 3D training, better selection and preparation of patients, improvement to the health plan including a different format and greater patient

For peer review only

ownership, considering greater flexibility in follow-up reviews that might include greater intensity of follow-up for selected patients, and evaluating implementation over a longer period (although that clearly has significant cost implications). If delivered as a whole practice intervention outside the context of a research trial, it is likely that implementation could improve and could lead to a more effective intervention. However, this creates the paradox that providing an intervention outside the context of research is more likely to provide a true representation of its effectiveness, but this cannot be proved without research. For example, the NHS Year of Care model is consistent with the principles of 3D, and has similar intentions, and has been iteratively developed and implemented over at least 10 years by the NHS Year of Care organisation. Promising results have been reported in pilot evaluations of this model, but it has not been subject to a randomised controlled trial [39].

Conclusions

In the context of an intervention that followed the recommendations and best evidence for care of people with multimorbidity, where the trial provided strong evidence that there was no effect on the primary outcome of HRQoL but an improvement in patient-centred outcomes, we found evidence of both implementation and intervention failure. Although this challenges the assumption that implementation and intervention failure can be clearly distinguished, we believe that the distinction does provide a useful framework to help interpret trial findings and to systematically identify modifiable and non-modifiable factors to inform future implementation decisions. This paper provides a worked example of how to use these concepts in process evaluation. We conclude that in the case of the 3D trial a truer test of the intervention effectiveness might be achieved by modifications that support better implementation, including whole practice implementation over a longer period to allow embedding. It is important to examine reasons for implementation deficiencies to determine not only whether there were implementation failures but also the reasons for them and whether they might be modifiable in order to avoid discarding a potentially effective intervention.

For peer review only - http://bmjopen.bmj.com/site/about/guidelines.xhtml

Ethics approval

The trial and process evaluation were approved by the South-West England NHS Research Ethics Committee (14/SW/0011)

Consent for publication

Not applicable as all data have been anonymised data

Availability of data and materials

Data supporting the conclusions of this article are included within the article. The full qualitative and quantitative datasets used and/or analysed during the current study are available from the corresponding author on reasonable request.

Competing interests

The authors declare that they have no competing interests

Funding

This project was funded by the National Institute for Health Research Health Services and Delivery Research Programme (project number 12/130/15). The views expressed are those of the author(s) and not necessarily those of the NIHR or the Department of Health and Social Care.

Authors contributions:

CM, AS and BG designed the process evaluation. CS led the design of the 3D intervention and the randomised trial. CM collected and analysed the qualitative data with input from AS, LW and BG and led the analysis and write-up of the results presented in this paper. AS, BG and CS critically revised

For peer review only

the manuscript. KC helped to design the template, analysed the quantitative data it recorded and helped to collect administrative survey data. M-SM contributed to the design of the process evaluation and facilitated data collection in the role of trial manager. All authors discussed findings, commented on the paper and approved the final version.

Acknowledgements:

The authors sincerely thank all those practices, staff and patients who took part in the 3D trial and especially those who contributed to the process evaluation by consenting to interviews and observation. We thank Sue Cooke and Polly Duncan who collected and helped with the analysis of some of the qualitative data in the lead trial site, Rebecca Robinson who supported the process evaluation with data collection in the early stages, Daisy Gaunt who did the main quantitative analysis for the trial, and the researchers in supporting trial sites, Bridie FitzPatrick, John McLeod, Caroline Gardner, Victoria Lee and Keith Moffat who supported the process evaluation by facilitating data collection and collecting administrative survey data. We also thank Pete Bower and Stewart Mercer, the Principle Investigators at those sites, who contributed to trial team discussions on the process evaluation design and findings. We also acknowledge other members of the wider trial team, Sandra Hollinghurst, Bryar Kadir, Emma Moody, Imran Rafi, and Joanna Thorn. Finally, we thank the Patient Public Involvement group who contributed valuable insight into qualitative findings and great enthusiasm for the study.

For peer review only - http://bmjopen.bmj.com/site/about/guidelines.xhtml

Table 1: Data from intervention practices used for this study

Data	Practices (N=16)	Data collected from:	Data used to examine:
Electronic data capture	All	3D electronic template recording of reviews completed and review components delivered to all patients	Reach and maintenance<br>Fidelity of delivery of intervention components to patients
Administrative survey	All	Research team completed questionnaire about organisation of reviews in all intervention practices	Adoption, reach and maintenance
Baseline interviews	4	4 administrators, 4 nurses, 5 GPs	Individual practice context to understand adoption and reach.
3D review observations	8	13 nurses, 15 GPs, 22 patients[1]	Variation in delivery of intervention components to patients
Post review debriefs and informal interviews	8	12 nurses, 7 GPs, 10 patients	Variation in delivery of intervention components to patients<br>Maintenance of intervention delivery
Patient focus groups	4	22 patients[2]	Variation in delivery of intervention components to patients
End-of trial interviews	5	4 administrators, 6 nurses, 5 GPs, 7 patients	Variation in delivery of intervention components to patients.<br>Maintenance of intervention delivery

1. 6 patients were observed for both parts of review
2. 2 focus groups of 3 patients, 1 focus group of 7 patients and 1 focus group of 7 patients and 2 carers

For peer review only

For peer review only - http://bmjopen.bmj.com/site/about/guidelines.xhtml

Table 2: Intervention practices

Practice	Practice size	Combined reviews at baseline[1]	Admin involvement	3D review organisation[3]	Reach	Qualitative data collection[4]
Lovell	4,000 patients<br>4 GPs, 2 nurses	All combined	1 administrator. All aware	Appointment sent, review appointments paired	First review 94%<br>Second review 93%	In depth. All elements
Tothill	10,000 patients<br>40 GPs, 4 nurses	Some combined	All	Appointment sent, review appointments separate	First review 92%<br>Second review 86%	None
Macready	6,000 patients<br>6 GPs, 2 nurses	All combined	1 administrator. All aware	Appointment sent, review appointments paired	First review 92%<br>Second review 50%	Observation and post-review informal interview
Dunbar	15,000 patients<br>16 GPs, 5 nurses	All combined	All	Letter inviting patient to call, review appointments paired	First review 90%<br>Second review 75%	None
Cabot	10,000 patients<br>12 GPs, 5 nurses	Some combined	Research nurse only	Appointment sent, review appointments separate	First review 83%<br>Second review 74%	Observation and post-review informal interview
Beddoes	5,500 patients,<br>4 GPs, 3 nurses	All combined	All	Letter inviting patient to call, review appointments separate	First review 80%<br>Second review 82%	In depth. All elements
Guppy	8,000 patients<br>6 GPs, 3 nurses	All combined	1 administrator. All aware	Appointment sent, review appointments paired	First review 80%<br>Second review 76%	Observation and post-review informal interview
Penn	10,500 patients<br>9 GPs, 3 nurses	Some combined	1 administrator. All aware	Phone call to patient, review appointments paired	First review 80%<br>Second review 47%	None
Harvey	15,000 patients<br>13 GPs, 4 nurses	Some combined	All	Appointment sent, review appointments sometimes separate	First review 77%<br>Second review 44%	In depth All elements
Priestman	13,500 patients<br>10 GPs, 3 nurses	All combined	All	Letter inviting patient to call, review appointments paired	First review 75%<br>Second review 45%	None
Sharples	4,500 patients<br>4 GPs, 2 nurses	None combined	All	Letter inviting patient to call, review appointments separate	First review 71%<br>Second review 67%	None
Martineau	5,000 patients<br>4 GPs, 2 nurses	Some combined	2 administrators. Others unaware	Phone call to patient, review appointments paired	First review 69%<br>Second review 53%	None
Carpenter	14,500 patients<br>12 GPs, 4 nurses	All combined	Unsure if all aware	Letter inviting patient to call, review appointments paired	First review 67%<br>Second review 50%	Observation and post-review informal interview
Blackwell	13,500 patients<br>9 GPs, 7 nurses	All combined	Nurse and administrator. Others unaware.	Letter inviting patient to call, nurse completed both parts of review	First review 66%<br>Second review 9%	End of trial interviews
Davy	14,500 patients<br>12 GPs 5 nurses	Some combined	2 administrators. Others unaware	Appointment sent, later review appointments separate	First review 38%<br>Second review 0%	In depth. All elements

(1) Combined reviews means reviews were purposely arranged to include all long-term conditions where there was a nurse-led clinic. (2) Continuity of care based on visit entropy score; lower scores indicate greater continuity: High<50, Medium 50-60, Low>60. (3) Paired means that nurse and GP appointments made at the same time but could take place on different days. (4) See table 1 for details of qualitative data collected.

For peer review only - http://bmjopen.bmj.com/site/about/guidelines.xhtml

Table 3: Quantitative evaluation of reach

	No (%) of 3D reviews delivered
Practice level analysis	N= 16 practices
Reach (% expected number of reviews delivered)	
First review	Median 66% (range 38-94%)
Second review	Median 47% (range 0-93%)
Patient level analysis	N= 797
Delivery of 3D nurse and GP reviews[a]	
Two 3D reviews with both GP and nurse (full)	390 (49%)
One 3D review with both GP and nurse (partial)	205 (26%)
Other (eg nurse review but no GP review) (partial)	31 (4%)
No 3D reviews (none)	171 (21%)

[a] 622 (78%) patients had at least one nurse review; 599 (75%) had at least one GP review. 390 (49%) patients received a 'full' intervention (defined as having two reviews, with each review involving a nurse and a GP appointment which could be on the same day or different days i.e. four appointments in total) in the 15 months of follow-up. 21% received no intervention.

Table 4: Quantitative evaluation of component delivery

	No (%) of each element of the 3D review delivered
Delivery of pharmacist medication review	607/797 (76%)
For those with at least one GP or nurse review	
Most important problem noted (patient agenda)[1]	616/622 (99%)
EQ5D pain question noted (Quality of life)[1]	611/622 (98%)
PHQ9 depression screening noted[1]	599/622 (96%)
Patient agenda printed[1]	579/622 (93%)
Medication adherence noted[2]	506/599 (84%)
First patient problem noted[2]	590/599 (98%)
Noted 'what patient can do' for first problem (health plan)[2]	559/599 (93%)
Noted 'what GP can do' for first problem (health plan)[2]	554/599 (92%)
3D health plan printed[2]	461/599 (77%)

[1] Components delivered in the nurse part of the review of which 622 took place. If one patient had two reviews, this component was delivered in at least one

[2] Components delivered in the GP part of the review of which 599 took place. If one patient had two reviews, this component was delivered in at least one

For peer review only - http://bmjopen.bmj.com/site/about/guidelines.xhtml

References

1. National Institute for Health and Care Excellence. Multimorbidity: clinical assessment and management (NG56) [PDF]. London UK: National Institute for Health and Care Excellence (NICE); 2016 [Available from: https://www.nice.org.uk/guidance/ng56].
2. American Geriatrics Society Expert Panel on the Care of Older Adults with Multimorbidity. Patient-centered care for older adults with multiple chronic conditions: a stepwise approach from the American Geriatrics Society: American Geriatrics Society Expert Panel on the Care of Older Adults with Multimorbidity. Journal of the American Geriatrics Society. 2012;60(10):1957-68.
3. Muth C, van den Akker M, Blom JW, Mallen CD, Rochon J, Schellevis FG, et al. The Ariadne principles: how to handle multimorbidity in primary care consultations. BMC medicine. 2014;12.
4. Man MS, Chaplin K, Mann C, Bower P, Brookes S, Fitzpatrick B, et al. Improving the management of multimorbidity in general practice: protocol of a cluster randomised controlled trial (The 3D Study). BMJ open. 2016;6(4):e011261.
5. O'Cathain A, Thomas KJ, Drabble SJ, Rudolph A, Hewison J. What can qualitative research do for randomised controlled trials? A systematic mapping review. BMJ open. 2013;3(6).
6. Oakley A, Strange V, Bonell C, Allen E, Stephenson J, Team RS. Process evaluation in randomised controlled trials of complex interventions. British Medical Journal. 2006;332(7538):413-6.
7. Carroll C, Patterson M, Wood S, Booth A, Rick J, Balain S. A conceptual framework for implementation fidelity. Implementation science : IS. 2007;2:40.
8. Rychetnik L, Frommer M, Hawe P, Shiell A. Criteria for evaluating evidence on public health interventions. Journal of epidemiology and community health. 2002;56(2):119-27.
9. Greenhalgh T, Papoutsi C. Studying complexity in health services research: desperately seeking an overdue paradigm shift. BMC medicine. 2018;16(1):95.
10. Moore G, Audrey S, Barker M, Bond L, Bonell C, Hardeman W, et al. Process evaluation of complex interventions. UK Medical Research Council (MRC) guidance. MRC Population Health Science Research Network; 2014 2014.
11. Chaplin K, Bower P, Man MS, Brookes ST, Gaunt D, Guthrie B, et al. Understanding usual care for patients with multimorbidity: baseline data from a cluster-randomised trial of the 3D intervention in primary care. BMJ open. 2018;8(8):e019845.
12. Salisbury C, Man M-S, Bower P, Guthrie B, Chaplin K, Gaunt DM, et al. Management of multimorbidity using a patient-centred care model: a pragmatic cluster-randomised trial of the 3D approach. The Lancet. 2018;392(10141):41-50.
13. Salisbury C, Man MS, Chaplin K, Mann C, Bower P, Brookes S, et al. A patient-centred intervention to improve the management of multimorbidity in general practice: the 3D RCT. Health Services and Delivery Research: NIHR. 2019;7.
14. Mann C, Shaw A, Wye L, Salisbury C, Guthrie B. A computer template to enhance patient-centredness in multimorbidity reviews: a qualitative evaluation in primary care. The British journal of general practice : the journal of the Royal College of General Practitioners. 2018;68(672):e495-e504.
15. Curran GM, Bauer M, Mittman B, Pyne JM, Stetler C. Effectiveness-implementation hybrid designs: combining elements of clinical effectiveness and implementation research to enhance public health impact. Medical care. 2012;50(3):217-26.

For peer review only - http://bmjopen.bmj.com/site/about/guidelines.xhtml

16. Mann C, Shaw A, Guthrie B, Wye L, Man MS, Hollinghurst S, et al. Protocol for a process evaluation of a cluster randomised controlled trial to improve management of multimorbidity in general practice: the 3D study. BMJ open. 2016;6(5):e011260.
17. Hoffmann TC, Glasziou PP, Boutron I, Milne R, Perera R, Moher D, et al. Better reporting of interventions: template for intervention description and replication (TIDieR) checklist and guide. British Medical Journal. 2014;348:g1687.
18. Mann C, Chilcott S, Plumb K, Brooks E, Man MS. Reporting and appraising the context, process and impact of PPI on contributors, researchers and the trial during a randomised controlled trial - the 3D study. Res Involv Engagem. 2018;4:15.
19. Grant A, Treweek S, Dreischulte T, Foy R, Guthrie B. Process evaluations for cluster-randomised trials of complex interventions: a proposed framework for design and reporting. Trials. 2013;14:15.
20. Wells M, Williams B, Treweek S, Coyle J, Taylor J. Intervention description is not enough: evidence from an in-depth multiple case study on the untold role and impact of context in randomised controlled trials of seven complex interventions. Trials. 2012;13:95.
21. Ovretveit J. Understanding the conditions for improvement: research to discover which context influences affect improvement success. BMJ quality & safety. 2011;20 Suppl 1:i18-23.
22. May CR, Mair FS, Dowrick CF, Finch TL. Process evaluation for complex interventions in primary care: understanding trials using the normalization process model. BMC Family Practice. 2007;8:42.
23. Murray E, Treweek S, Pope C, MacFarlane A, Ballini L, Dowrick C, et al. Normalisation process theory: a framework for developing, evaluating and implementing complex interventions. BMC medicine. 2010;8:63.
24. Tong A, Sainsbury P, Craig J. Consolidated criteria for reporting qualitative research (COREQ): a 32-item checklist for interviews and focus groups. Int J Qual Health Care. 2007;19(6):349-57.
25. Sandelowski M. Whatever happened to qualitative description? Res Nurs Health. 2000;23(4):334-40.
26. Braun V, Clarke V. Using thematic analysis in psychology. Qualitative research in psychology. 2006;3(2).
27. Bice TW, Boxerman SB. A quantitative measure of continuity of care. Medical care. 1977;15(4):347-9.
28. Hawe P, Shiell A, Riley T. Complex interventions: how "out of control" can a randomised controlled trial be? British Medical Journal. 2004;328(7455):1561-3.
29. Basch CE, Sliepcevich EM, Gold RS, Duncan DF, Kolbe LJ. Avoiding type III errors in health education program evaluations: a case study. Health Educ Q. 1985;12(4):315-31.
30. Campbell NC. Designing and evaluating complex interventions to improve healthcare. British Medical Journal. 2007;334:455-9.
31. Kennedy A, Rogers A, Chew-Graham C, Blakeman T, Bowen R, Gardner C, et al. Implementation of a self-management support approach (WISE) across a health system: a process evaluation explaining what did and did not work for organisations, clinicians and patients. Implementation science : IS. 2014;9:129.
32. Bamford C, Poole M, Brittain K, Chew-Graham C, Fox C, Iliffe S, et al. Understanding the challenges to implementing case management for people with dementia in primary care in England: a qualitative study using Normalization Process Theory. BMC health services research. 2014;14:549.

For peer review only - http://bmjopen.bmj.com/site/about/guidelines.xhtml

33. Grant A, Dreischulte T, Guthrie B. Process evaluation of the Data-driven Quality Improvement in Primary Care (DQIP) trial: case study evaluation of adoption and maintenance of a complex intervention to reduce high-risk primary care prescribing. BMJ open. 2017;7(3):e015281.
34. Jansen YJ, de Bont A, Foets M, Bruijnzeels M, Bal R. Tailoring intervention procedures to routine primary health care practice; an ethnographic process evaluation. BMC health services research. 2007;7:125.
35. Bunce AE, Gold R, Davis JV, McMullen CK, Jaworski V, Mercer M, et al. Ethnographic process evaluation in primary care: explaining the complexity of implementation. BMC health services research. 2014;14(607):607.
36. Kennedy A, Chew-Graham C, Blakeman T, Bowen A, Gardner C, Protheroe J, et al. Delivering the WISE (Whole Systems Informing Self-Management Engagement) training package in primary care: learning from formative evaluation. Implementation science : IS. 2010;5(1):7.
37. Kennedy A, Rogers A, Bowen R, Lee V, Blakeman T, Gardner C, et al. Implementing, embedding and integrating self-management support tools for people with long-term conditions in primary care nursing: a qualitative study. International journal of nursing studies. 2014;51(8):1103-13.
38. Eaton S, Roberts S, Turner B. Delivering person centred care in long term conditions. British Medical Journal. 2015;350:h181.
39. Year of Care. Report of findings from the pilot programme. 2011.

For peer review only

For peer review only - http://bmjopen.bmj.com/site/about/guidelines.xhtml

Figure 1: 3D intended intervention work and core components

Adoption by the practice – intended administrative activity

- Identify patients with ≥ 3 long-term conditions and flag on EMIS
- Install purpose-designed electronic 3D review template
- In consultation with clinicians, allocate a named GP (and nurse if appropriate) for all reviews
- All appointments outside reviews scheduled with named GP and/or nurse and offered as longer appointments
- Schedule participating patients for 6 monthly 3D review of all conditions together in extended two-part appointments, first part with named nurse, second part with named GP
- Cancel usual long-term condition reviews, and replace with 3D review
- Run monthly monitoring searches and send them to researchers

Core components

- Continuity of care
- A comprehensive review arranged with named nurse and GP in separate appointments every six months
- Longer appointments with named GP or nurse as needed between reviews

3D multimorbidity reviews – intended GP, nurse and pharmacist activity

- Two-part long-term condition review with named nurse and GP, to address all conditions together, using new 'intelligent' 3D review template.
- Part 1 typically done by a nurse: identify patient's priorities and quality of life issues, screen for depression and complete disease checks. Create agenda for second part of review based on this information and give printed copy to patient.
- Pharmacist review of medication prior to part 2
- Part 2 typically done by a GP: address agenda, review treatment and medication adherence, aim to optimise medication and reduce treatment burden, agree health plan with patient and provide written copy
- Involvement of secondary care physician if needed

Core components

- Compile patient agenda based on patient priorities and clinical measures and provide copy to patient
- Depression screening
- Attention to quality of life
- Chronic disease monitoring
- Medication review and adherence
- Share printed health plan with actions for both patient and GP

For peer review only - http://bmjopen.bmj.com/site/about/guidelines.xhtml

Figure 2: Process evaluation design and research questions (research stages addressed in this paper are shown in solid blue)

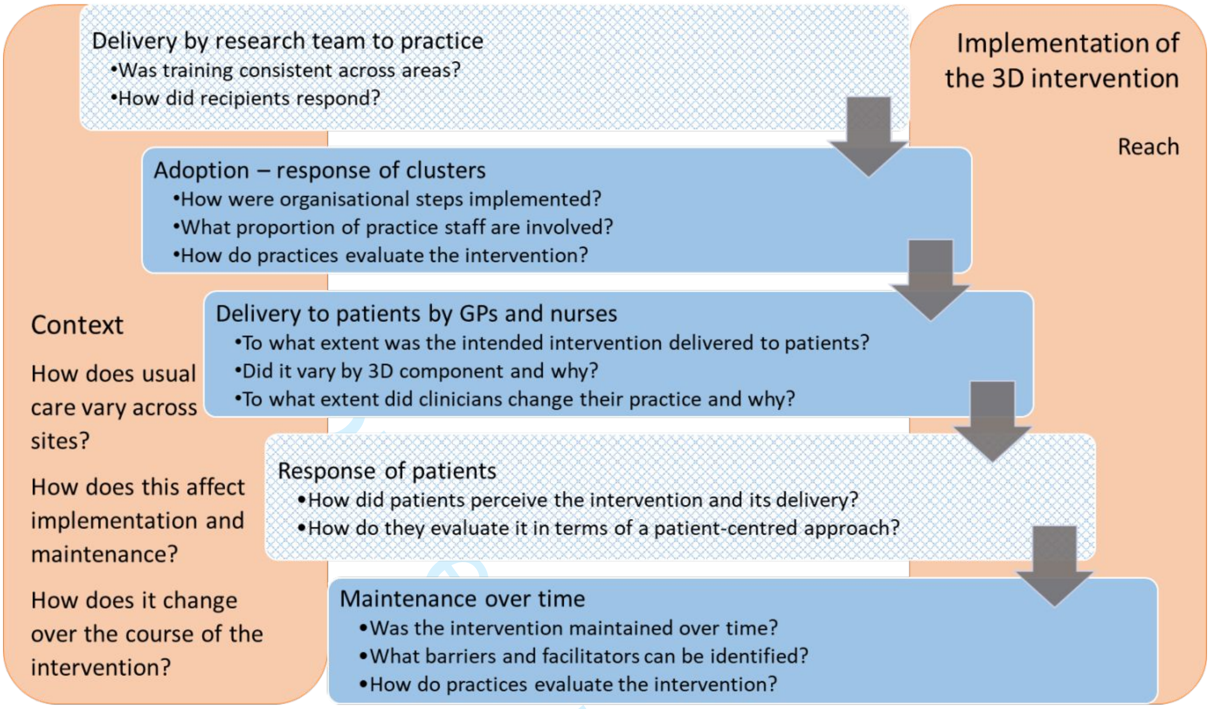

For peer review only

Appendix 1: Tidier checklist for the 3D intervention

Additional information can be found in the published full report of the trial: Salisbury C, Man M-S, Chaplin K, Mann C, Bower P, Brookes S, et al. A patient-centred intervention to improve the management of multimorbidity in general practice: the 3D RCT. Health Serv Deliv Res 2019;7(5)

Item No	Item		Summary information and location of full detail in report
**Brief name**			
1	Provide the name or a phrase that describes the intervention	✔	Improving the management of multimorbidity in general practice – the 3D study
**Why**			
2	Describe any rationale, theory, or goal of the elements essential to the intervention	✔	Underlying theoretical basis is the Patient-centred Care Model. Intervention designed to address problems experienced by people with multimorbidity and aimed to achieve improved quality of life.<br>*Report Pages 3, 9*
**What**			
3	Materials: Describe any physical or informational materials used in the intervention, including those provided to participants or used in intervention delivery or in training of intervention providers. Provide information on where the materials can be accessed (such as online appendix, URL)	✔	An purpose-designed IT template was used within Egton Medical Information Systems (EMIS) which when completed generated a patient agenda and a patient health plan. Intervention patients received a 3D card which identified them to practices and specified their named GP.<br>*Report Pages 11-15 and Appendices 3, 5-8*<br>*Report Supplementary Material 1 and 2*
4	Procedures: Describe each of the procedures, activities, and/or processes used in the intervention, including any enabling or support activities	✔	This was highly complex intervention that incorporated:<br>Installing the EMIS template<br>Identifying and recruiting the target group<br>Allocating a named GP and nurse for each participant and issuing a 3D card to each participant to improve continuity of care.<br>Training the practice staff and clinicians<br>Organising and delivering 6 monthly 3D comprehensive reviews of all health conditions and of psychosocial factors that were delivered in 2 parts, first with the named nurse, second with the named GP.<br>Medication review by pharmacist viewing patient record remotely<br>Meetings of practice champions<br>Provision of monthly monitoring feedback to practices about their delivery of the intervention<br>*Report pages 10 -15*

For peer review only

Item No	Item		Summary information and location of full detail in report
**Who provided**			
5	For each category of intervention provider (such as psychologist, nursing assistant), describe their expertise, background, and any specific training given	✔	Intervention providers included GPs, nurses in general practice, pharmacists, general practice administrators and receptionists, and one secondary care physician for each area.<br>*Report page 12*
**How**			
6	Describe the modes of delivery (such as face to face or by some other mechanism, such as internet or telephone) of the intervention and whether it was provided individually or in a group	✔	Face-to-face delivery of comprehensive 6 monthly reviews. Remote performance of medication review element<br>*Report pages 11-15*
**Where**			
7	Describe the type(s) of location(s) where the intervention occurred, including any necessary infrastructure or relevant features	✔	The intervention occurred in individual general practices in three areas of the UK
**When and How Much**			
8	Describe the number of times the intervention was delivered and over what period of time including the number of sessions, their schedule, and their duration, intensity, or dose	✔	The intervention two-part reviews were delivered twice in 12 months. The intervention components were mainly delivered in these reviews carried out in nurse appointments of 30-50 minutes and in GP appointments of 20<br>*Report pages 12-13*
**Tailoring**			
9	If the intervention was planned to be personalised, titrated or adapted, then describe what, why, when, and how	✔	Practices were allowed some flexibility in how intervention delivery was organised<br>*Report page 14*
**Modifications**			
10	If the intervention was modified during the course of the study, describe the changes (what, why, when, and how)		The intervention was modified after piloting from a whole practice service change intervention to selected patients only.<br>*Report page 16 and Appendix 14*
**How well**			
11	Planned: If intervention adherence or fidelity was assessed, describe how and by whom, and if any strategies were used to maintain or improve fidelity, describe them	✔	Mixed methods were used involving both quantitative and qualitative researchers in the trial team. Quantitative methods involved electronic monitoring of delivery of intervention components. Qualitative

For peer review only

Item No	Item		Summary information and location of full detail in report
			methods included interviewing participants and providers and observing delivery. Strategies to maintain and improve fidelity were the monthly electronic monitoring feedback, meetings of practice champions and financial incentives *Report pages 31-33*
12	Actual: If intervention adherence or fidelity was assessed, describe the extent to which the intervention was delivered as planned	✔	Half the participants received the full intended number of reviews. In delivered reviews most components were delivered but the way they were delivered varied. This is presented and discussed in the conclusion of the present paper. *Report pages 77-86*

For peer review only

Appendix 2

Consolidated criteria for reporting qualitative studies (COREQ): 32-item checklist

1.	Interviewer/facilitator	The interviews, focus groups and observations were conducted by Cindy Mann, with the exception of 5 observations and one interview that were carried out by Polly Duncan.
2.	Credentials	Cindy Mann had an MSc and previous qualitative research experience at the time of the study. Polly Duncan is an academic GP and was new to qualitative research at that time
3.	Occupation	CM was a senior research associate, Polly Duncan was a GP with an academic training fellowship
4.	Gender	Both female
5.	Experience and Training	CM has training in qualitative research and research methods. Experience in various environments (primary care and secondary care) as a researcher, research nurse and clinical nurse. Experience as a counsellor and group facilitator. PD is a qualified GP with additional academic experience of research
6.	Relationship established	Prior to study commencement, the interviewer and the participants had no previous contacts. Rapport was built before interview, focus groups or observations by answering questions from participants and taking informed consent.
7.	Participant knowledge of the interviewer	The participants did not have prior knowledge of the interviewer before the study. When participants were recruited, they were provided with an information leaflet about the study and purpose of the interview/focus group/observation which was repeated prior to data collection beginning. Information about the researcher was not provided other than her role in the research team.
8.	Interviewer characteristics	The main researcher was a white, university-educated British woman with a nursing, counselling and research background. Qualitative research is always influenced by the perspective of the researcher, and in this case the nursing perspective and primary care clinical experience may have fed into the way some clinical participants were

For peer review only

	interviewed. Since the purpose of this paper is not primarily to report the findings of a qualitative piece of research but rather the findings of a process evaluation of a complex intervention and the fidelity with which it was implemented, some of the usual detail for reporting qualitative research has not been included in the manuscript.
9. Methodological orientation and Theory	The key methodological framework used was a framework for process evaluation for cluster randomised trials and the MRC guidance for the process evaluation for complex interventions framework. Mixed methods were used, and thematic analysis was used for the qualitative data.
10. Sample	Practices taking part in the process evaluation were purposively sampled based on their characteristics. Individual staff members and clinicians of those practices who were taking part in the trial were invited to take part in the process evaluation. Patient participants were sampled based on their responses to a baseline questionnaire
11. Method of approach	Patient participants were approached by invitation letter including information sheet and staff and clinicians by email with invitation letter and information attached. In both cases follow up contact was made to discuss possible participation.
12. Sample Size	The total number of interviews with staff, including informal debriefs after 3D reviews, was 32 (18 GPs, 20 nurses and 9 administrator interviews). Some individuals were interviewed twice so the actual number of those interviewed was 11 GPs, 14 nurses, 7 administrators and 38 patients (including the 22 patients who attended a focus group). 28 intervention review observations were carried out.
13. Non-participation	Some patients refused interviews or focus group and 1 nurse refused review observation
14. Setting of Data Collection	Interviews were conducted in GP practices, patients' homes or, in the case of focus groups, local halls, depending on convenience and patient preference. Observations were all carried out at the GP practice.

For peer review only

15. Presence of non-participants	Patients' carers were sometimes present at review observations, interviews or focus groups but all of them also provided consent. The researcher was present in a non-participatory role at observations
16. Description of the sample	GPs, administrators, practice nurses and patients from 9 different GP practices
17. Interview guide	Interview guides, a focus group schedule and an observation guide were used to act as a checklist but without imposing a set structure
18. Repeat interviews	Repeat interviews were carried out with some nurses, GPs and administrators who were interviewed both at beginning and end of the trial
19. Audio-/visual recording	We used audio recording to collect all data.
20. Field notes	Field notes were made during the observations to note participant expression, or other non-verbal cues and in all instances of data collection to describe the ambience of the GP practice and reception and aspects of the environment and interaction.
21. Duration	The interviews lasted between 5 and 50 minutes. Focus groups lasted an hour. Review observations lasted between 20 and 60 minutes.
22. Data Saturation	The concept of information power was used but is less relevant to this manuscript because of the process evaluation focus and has not therefore been reported
23. Transcripts returned	Transcripts were not returned to participants for comment or correction.
24. Number of data coders	One (Cindy Mann), with double coding of a sub-sample by Alison Shaw, Lesley Wye, Polly Duncan and 2 members of the Patient Public Involvement group
25. Description of the coding tree	Not included in this manuscript because the purpose of this paper is not primarily to report the findings of a qualitative piece of research
26. Derivation of themes	As above. Themes in the qualitative were a priori based on intervention components, supplemented by themes arising from the data
27. Software	NVivo v11

For peer review only

28. Participant checking	No
29. Quotations presented	Yes, participant quotations are presented to illustrate the themes.
30. Data and findings consistent	Yes.
31. Clarity of major themes	Major themes are based around intervention components as the purpose of the paper is to assess implementation fidelity
32. Clarity of minor themes	Not applicable

For peer review only

Additional file 3: Electronic monitoring of review component delivery

Practice	Penn	Priestman	Sharples	McReady	Harvey	Blackwell	Guppy	Lovell	Tothill	Beddoes	Dunbar	Plimsoll[1]	Carpenter	Davy	Cabot	Martineau	ALL
**3d agenda printed**	97%	92%	100%	89%	**97%**	81%	95%	**98%**	98%	**100%**	100%	70%	97%	**58%**	100%	92%	**96%**
**3d health plan printed**	77%	81%	97%	91%	**62%**	31%	23%	**100%**	80%	**98%**	85%	39%	85%	**80%**	98%	67%	**83%**
**adherence meds**	95%	61%	94%	96%	**65%**	92%	63%	**100%**	39%	**67%**	62%	44%	54%	**50%**	93%	64%	**71%**
**EQ5D pain**	47%	97%	100%	71%	**100%**	96%	65%	**52%**	100%	**98%**	100%	5%	100%	**100%**	100%	95%	**83%**
**GP first goal noted**	100%	97%	100%	100%	**76%**	96%	100%	**100%**	102%	**98%**	102%	44%	100%	**95%**	93%	97%	**94%**
**Most important problem on nurse view**	100%	97%	100%	100%	**100%**	96%	100%	**100%**	100%	**100%**	100%	100%	100%	**100%**	100%	97%	**99%**
**Pharmacist comment**	84%	100%	95%	82%	**105%**	78%	83%	**100%**	107%	**106%**	100%	38%	78%	**43%**	100%	102%	**88%**
**Pharmacist comments noted?**		56%	53%		**47%**	77%			68%	**69%**	92%		56%	**80%**	95%	64%	**69%**
**PHQ9 done**	97%	97%	100%	91%	**91%**	96%	98%	**100%**	98%	**98%**	100%	100%	97%	**94%**	100%	103%	**98%**
**what GP can do about main problem**	92%	89%	100%	98%	**71%**	73%	98%	**76%**	100%	**89%**	102%	33%	87%	**80%**	90%	72%	**84%**
**what patient can do about main problem noted**	77%	92%	86%	96%	**76%**	73%	100%	**91%**	100%	**93%**	100%	39%	97%	**85%**	90%	78%	**86%**
**3D participants Pharmacist comment**	84%	100%	95%	82%	**105%**	78%	83%	**100%**	107%	**106%**	100%	38%		**43%**	100%	102%	**88%**

Key: Range of fidelity from red (worst) to green (best)
Grey-shaded column headers indicate case study practices
Practice ID: 60 = Harvey; 46 = Lovell; 26 = Beddoes; 69 = Davy
Some values are greater than 100% because percentages were calculated based on the number of participants remaining in the trial at the end
[1]This practice stopped delivering the intervention and withdrew from the process evaluation

BMJ Open

Can implementation failure or intervention failure explain the result of the 3D multimorbidity trial in general practice: mixed methods process evaluation

Journal:	*BMJ Open*
Manuscript ID	bmjopen-2019-031438.R1
Article Type:	Original research
Date Submitted by the Author:	06-Aug-2019
Complete List of Authors:	Mann, Cindy; University of Bristol School of Population Health Sciences, Centre for Academic Primary Care<br>Shaw, Ali; University of Bristol School of Population Health Sciences, Centre for Academic Primary Care<br>Guthrie, Bruce; University of Edinburgh College of Medicine and Veterinary Medicine,<br>Wye, Lesley; University of Bristol, Centre for Academic Primary Care<br>Man, Mei-See; University of Bristol School of Population Health Sciences, Centre for Academic Primary Care<br>Chaplin, Katherine ; University of Bristol Centre for Academic Primary Care, Bristol Medical School<br>Salisbury, Chris; University of Bristol School of Population Health Sciences, Centre for Academic Primary Care
**Primary Subject Heading**:	General practice / Family practice
Secondary Subject Heading:	Health services research, Patient-centred medicine, Research methods
Keywords:	Process evaluation, Multimorbidity, Implementation fidelity, Patient-centred, Intervention failure, PRIMARY CARE

SCHOLARONE™
Manuscripts

Can implementation failure or intervention failure explain the result of the 3D multimorbidity trial in general practice: mixed methods process evaluation

Cindy Mann, Alison Shaw, Bruce Guthrie, Lesley Wye, Mei-See Man, Katherine Chaplin, Chris Salisbury

Cindy Mann[1], cindy.mann@bristol.ac.uk **Corresponding author**

Alison Shaw[1], ali.heawood@bristol.ac.uk

Bruce Guthrie[2], bruce.guthrie@ed.ac.uk

Lesley Wye[1], lesley.wye@bristol.ac.uk

Mei-See Man[1], mei-see.man@bristol.ac.uk

Katherine Chaplin[1], k.chaplin@bristol.ac.uk

Chris Salisbury[1], c.salisbury@bristol.ac.uk

[1]Centre for Academic Primary Care, NIHR School for Primary Care Research, Population Health Sciences, Bristol Medical School, University of Bristol, 39 Whatley Road, Bristol BS8 2PS, UK

[2] Usher Institute of Population Health Sciences and Informatics, College of Medicine and Veterinary Medicine, University of Edinburgh, Old Medical School, Teviot Place, Edinburgh, EH8 9AG, UK

For peer review only

For peer review only - http://bmjopen.bmj.com/site/about/guidelines.xhtml

Abstract

Objectives

During a cluster-randomised trial (The 3D Study) of an intervention enacting recommended care for people with multimorbidity, including continuity of care and comprehensive biennial reviews, we examined implementation fidelity to interpret the trial outcome and inform future implementation decisions.

Design

Mixed methods process evaluation using cross-trial data and a sample of practices, clinicians, administrators and patients. Interviews, focus groups and review observations, were analysed thematically and integrated with quantitative data about implementation. Analysis was blind to trial outcomes and examined context, intervention adoption, reach and maintenance, and delivery of reviews to patients.

Setting

Thirty-three UK general practices in three areas

Participants

The trial included 1546 people with multimorbidity. Eleven GPs, 14 nurses, 7 administrators and 38 patients from 9 of 16 intervention practices were sampled for interview.

Results

Staff loss, practice size and different administrative strategies influenced implementation fidelity. Practices with whole administrative team involvement and good alignment between the intervention and usual care generally implemented better. Fewer reviews than intended were delivered (49% of patients receiving both intended reviews, 30% partially reviewed). In completed reviews >90% of intended components were delivered but review observations and interviews with patients and clinicians found variation in style of component delivery, from 'tick-box' to patient-centred approaches. Implementation barriers included inadequate skills training to implement

For peer review only - http://bmjopen.bmj.com/site/about/guidelines.xhtml

For peer review only

patient-centred care planning, but patients reported increased patient-centredness due to comprehensive reviews, extra time and being asked about their health concerns.

Conclusions

Implementation failure contributed to lack of impact of the 3D intervention on the trial primary outcome (quality of life), but so did intervention failure since modifiable elements of intervention design were partially responsible. When a decisive distinction between implementation failure and intervention failure cannot be made, identifying potentially modifiable reasons for sub-optimal implementation is important to enhance potential for impact and effectiveness of a re-designed intervention.

Trial registration number

ISRCTN06180958 registered 18.2.2014

Key words

Process evaluation, implementation fidelity, intervention failure, implementation failure, multimorbidity, primary care, patient-centred, null trial

Strengths and limitations of this study:

- In the largest randomised controlled trial of a recommended patient-centred model of care for people with multimorbidity, we conducted a comprehensive process evaluation to examine implementation fidelity in case of a null result and to inform future implementation.
- We used mixed methods to evaluate multiple aspects of implementation and a wide range of factors that might influence implementation.

For peer review only - http://bmjopen.bmj.com/site/about/guidelines.xhtml

- Although distinguishing between implementation failure and intervention failure is recommended in null trials to avoid needlessly discarding a promising intervention, the distinction is difficult to apply when aspects of intervention design contribute to implementation deficiencies.
- By investigating reasons for implementation deficiencies, and distinguishing between potentially modifiable and non-modifiable reasons, we have instead provided information that is potentially more valuable than dichotomising between implementation failure and intervention failure for informing decisions about wider implementation, or the need for further research.

Introduction

The increasing prevalence of multimorbidity, driven by aging populations across the world, is a major challenge to health services. There is broad consensus about how primary care for people with multimorbidity should be organised [1-3], but little evidence about the effectiveness of recommended strategies. Reflecting this absence of evidence, the 2016 National Institute of Health and Care Excellence Multimorbidity clinical guideline recommended more research on how best to organise primary care to address the challenge of improving care for people with multimorbidity [3]. In the largest trial to date of an intervention based on the consensus of opinion about best practice for multimorbidity care, the 3D study evaluated a patient-centred approach for people with multimorbidity, defined for this trial as people with three or more long-term conditions on a disease registry. The approach included continuity of care and regular holistic reviews (3D reviews) in primary care (General Practices in the UK) with a focus on addressing quality of life, mental as well as physical health, and polypharmacy. The hypothesis was that this would improve patient-centred care, reduce treatment burden and illness burden and improve quality of life (the trial primary outcome) [4].

For peer review only - http://bmjopen.bmj.com/site/about/guidelines.xhtml

Process evaluation of trials evaluating complex interventions can inform decisions about the wider implementation and applicability of those interventions. A comprehensive process evaluation can help interpret trial results and inform real-world implementation [5, 6] by providing explanations when interventions are not effective [7]. This may be because of *intervention failure* (the intervention was delivered as intended but did not improve outcomes, so should not be implemented) and/or *implementation failure* (the intervention was inadequately implemented and so might need additional research to further examine effectiveness) [8]. However, distinguishing implementation and intervention failure is often not straightforward [9, 10] and may require detailed examination of implementation fidelity.

We have previously reported baseline data from the 3D Study [11], main trial findings [12, 13] and analysis of the patient-centredness of the 3D review [14]. At baseline, many practices had already combined multiple long-term condition reviews into one appointment but other recommended care [1, 3] was less evident. For example, only 10% of patients were aware of receiving a care plan and 35% were rarely or never asked what was important to them in managing their health [11]. The main trial results showed no effect from the 3D intervention on the primary outcome of health-related quality of life (HR-QOL) or other related secondary outcomes such as wellbeing and treatment burden, but a consistent beneficial effect on patients' experience of care as more person-centred [12]. Analysis of observational and interview data about intervention delivery indicated that the main reasons for the perceived increase in patient-centredness were that when patients attended for an intervention review, they were first asked about their most important health concerns and then given a longer, comprehensive review encompassing all health issues [14]. The aim of this paper is to examine whether the measured lack of effect on the primary outcome in the 3D trial was due to implementation or intervention failure, and thereby inform future intervention development and evaluation.

For peer review only - http://bmjopen.bmj.com/site/about/guidelines.xhtml

Methods

Setting - The 3D study

The intervention and trial evaluation are described briefly here, having been reported in detail elsewhere [4, 12, 13]. The core components of the intervention included offering greater continuity of care and six-monthly, two-part patient-centred, comprehensive health reviews, conducted by a named nurse and GP and underpinned by a purpose-designed electronic template (Figure 1). A pharmacist also completed an electronic medication review. Practices were expected to deliver two complete reviews to every patient during the trial, including all review components. However, practices could decide the detail of how they would provide the reviews, enhance continuity of care and reduce the number of review appointments. Administrators and clinicians nominated by the practices received two short (2-3 hours) training sessions from the trial team on the intervention's rationale and the use of the computer template. Appendix 1 shows the TIDieR checklist [15] for the intervention design. Figure 1 details the work that administrative staff, clinicians and pharmacists were expected to do to deliver the intervention. Sixteen general practices received the intervention compared to 17 control practices, with 1546 participating patients [4]. However, because of staffing crises, one intervention practice stopped delivering the intervention and withdrew from the process evaluation.

Patient and Public involvement

A patient public involvement group was set up during development of the trial intervention pre-funding to ensure that it met the perceived needs of people with multimorbidity. The group was actively involved throughout the trial in multiple ways, as reported by Mann et al. [16].

Process evaluation design

The design is briefly reported here as a detailed description is provided in our earlier paper [17]. We based the design on a process evaluation framework for cluster randomised trials [18], and also

For peer review only - http://bmjopen.bmj.com/site/about/guidelines.xhtml

considered UK Medical Research Council guidance for process evaluation of complex interventions [10]. This, rather than qualitative methodology criteria, underpins the rigour of the research as our focus was to ensure a comprehensive process evaluation that examined all aspects of intervention implementation that might affect the results of the trial. As such, the interview schedules were semi-structured to elicit specific information to answer the process evaluation research questions and the size of our qualitative sample was determined by information power [19] regarding implementation variation and the reasons for it, rather than data saturation.

We based the process evaluation on a logic map describing the intervention design and used the logic map to inform assessment of implementation fidelity (the extent to which practices implemented the intervention as the researchers intended) [17]. The assessment covered adoption of the 3D intervention (implementation of the organisational components of the intervention); delivery of 3D reviews to patients; maintenance (whether delivery is sustained over time) and reach (the number of participants who receive the intervention) (Figure 2), and the important influence of context on implementation fidelity, maintenance and reach [20-23].

Data collection

Qualitative data collection in selected practices

Intervention practices were sampled at different stages for qualitative data collection (Table 1). Four practices were initially purposefully sampled during early stages of the trial, using baseline data and observation of practice team training, for detailed qualitative investigation of all aspects of implementation, including context, adoption, delivery and maintenance. This sampling reflected our assumptions that (a) larger practices may have lower continuity of care and a lower proportion of clinicians taking part in 3D which may influence implementation; and (b) practices whose care for patients with multimorbidity already reflected aspects of the 3D approach may adopt 3D more readily. These four practices were included in every stage of data collection. An additional five practices were responsively sampled at later stages for focused observation of clinicians' style

For peer review only - http://bmjopen.bmj.com/site/about/guidelines.xhtml

147 of delivery of 3D reviews and to examine variations in models of delivery that emerged during the
148 trial. In total nine of the 16 intervention practices were sampled.

149 All intervention practices were given pseudonyms to preserve anonymity. Data collected included:
150 interviews with practice staff; non-participant observation of 3D reviews with follow-up interviews
151 with clinicians and patients; and focus groups and interviews with patients (Table 1), all of which
152 were audio-recorded. The qualitative data were almost all collected by CM, a female qualitative
153 researcher experienced in focus groups and interviews and with clinical nursing experience including
154 as a practice nurse. Five observations and one interview were carried out by a female GP gaining
155 experience in qualitative research (PD). The interview topic guides and observation checklist are
156 shown in Appendix 2. All the analysis was carried out by CM with support from BG, a GP, health
157 services researcher and process evaluation methodologist, and AH, a highly-experienced qualitative
158 and process evaluation researcher.

159 *Interviews with practice staff:* At baseline, interviews in the four initially-sampled practices with the
160 3D lead GP, the lead nurse and the key administrator explored usual care, initial reactions to the
161 intervention and implementation arrangements. Interviews at the end of the trial in the same four
162 practices, and in a fifth, responsively sampled practice where a nurse practitioner delivered all
163 reviews, explored experience of delivering the intervention and maintenance. These interviews
164 lasted 15-50 minutes. Most individuals were interviewed at both the beginning and end of the trial
165 to achieve a longitudinal perspective on implementation and to see how their initial response to the
166 intervention changed in light of their experience of implementing it, but there were also a few single
167 interviews.

168 *Observation of 3D reviews with follow-up interviews:* Twenty-eight 3D reviews were observed and
169 recorded in the four initially-sampled practices and in four of the responsively sampled practices,
170 including one in which a research nurse, rather than a practice nurse, conducted most of the part 1
171 reviews. Observation notes were informed by an observation checklist (Appendix 2). The checklist

For peer review only - http://bmjopen.bmj.com/site/about/guidelines.xhtml

was based on the intervention components and directed attention to whether components were delivered and the manner of their delivery. Where possible, brief follow-on interviews with the clinician and/or patient whose review had been observed were completed on the same day. These interviews lasted 5-24 minutes.

Focus groups and interviews with patients: In the four initially-sampled practices, patients varying in health status and satisfaction with care according to baseline questionnaire data were invited to focus groups or individual interview towards the end of the trial, to explore their experience of receiving the intervention. One focus group per practice took place, lasting about one hour. Patients preferring individual interviews were interviewed for 20-50 minutes in a convenient location, usually their own home. All the focus groups and interviews were carried out by CM.

Appendix 3 shows the COREQ checklist [24] for qualitative methodology and provides additional detail.

Quantitative data collected from all intervention practices

Data about 3D review completion were extracted each month from the routine electronic medical records to evaluate intervention reach, delivery and maintenance [4, 17]. The data included dates of reviews, who had completed the review, and whether core elements were recorded as delivered in the 3D review template. In the first part of the review delivered by a nurse, data included description of patients' main concerns, pain levels, depression screening, and the creation and printing of a patient agenda. The template also recorded the pharmacist's completion of a medication review, their recommendations and whether these had been noted by the GP. In the second part of the review delivered by a GP (except in one practice), recorded data included medication adherence and description of at least one main problem in the health plan, together with patient and GP actions to address the problem. Finally, the software recorded whether an agreed health plan had been printed.

For peer review only - http://bmjopen.bmj.com/site/about/guidelines.xhtml

Survey data collected in all intervention practices

Researchers in each trial area completed a purpose-designed administrative survey about the way 3D reviews were organised in all intervention practices. The survey included the proportion of the administrative team involved in 3D, how patients were identified and contacted, and whether practices facilitated 3D patients seeing their named GP at appointments other than 3D reviews.

Data analysis

All audio-recordings of qualitative data (interviews, focus groups and consultation recordings) were professionally transcribed, then the transcript was checked against the recording, anonymised and annotated with observation notes. The annotation process aided interpretation of the data and illuminated the manner of delivery in the recorded consultations. We applied qualitative description methodology to write individual accounts [25] of context and adoption of the intervention in the four practices initially sampled for detailed examination [13], and cross-case thematic analysis [26] to identify recurring issues relevant to intervention adoption, delivery and maintenance in all nine selected practices. The data were analysed in parallel with data collection, so that emerging issues were incorporated into future data collection. For the thematic analysis, NVivo v.11 software (QSR International) was used to facilitate both deductive coding derived from intervention components and inductive coding derived from the data [26], allowing the identification of both anticipated themes (e.g. those relating to the key components of the intervention) and emergent themes across sampled practices. Qualitative analysis was led by CM with input from AS, LW and BG, who commented on the developing coding framework, double-coded a sample of transcripts and agreed the final themes. Additionally, to further enhance trustworthiness and credibility of findings, two members of the Patient and Public Involvement group each coded four transcripts to check interpretation of the data from the patient perspective. Quantitative data were analysed descriptively by CM and KC and integrated with qualitative data.

For peer review only - http://bmjopen.bmj.com/site/about/guidelines.xhtml

All process evaluation data collection and analyses were done blind to the trial outcome, so that interpretation would not be influenced by knowing the results of the primary outcome.

Results

The results examine 1) Adoption of the intervention by practices, 2) Reach and maintenance, and 3) Delivery of reviews to patients. In quotes, staff and patients are identified by practice pseudonym, role and a number.

Adoption – organisational components

The two core components of organisational adoption were continuity of care and arranging the two-part 3D reviews.

Continuity of care

Practices were asked to allocate a named GP to 3D patients for their reviews and for any appointment between reviews. Continuity of care was evaluated as a secondary outcome for the trial and, measured using the Continuity of Care index [27], increased slightly in the intervention arm [12]. However, some patients experienced reduced continuity because their GP left during the trial. Others were allocated a different GP for the intervention, either to share work-load or because their usual GP was not participating in 3D. These patients often continued to see their usual GP for appointments other than reviews.

> *[My usual GP] had to get changed. There's three doctors in our practice and they were doing I think 12 patients, so it was split between three doctors. So I had to go with [GP2]. (Focus group Lovell Patient 8)*

The four initially-sampled practices (Beddoes, Davy, Harvey and Lovell) provided insight into contextual influences. Harvey already had a "personal list" system with high continuity, but during the trial this was disrupted when several GPs left the practice. Beddoes supported 3D participants to

For peer review only - http://bmjopen.bmj.com/site/about/guidelines.xhtml

see their allocated GP between reviews. At Davy, continuity was poorly implemented due to staff loss and because receptionists were unaware of 3D. Lovell continued with their usual system, which they felt delivered adequate continuity of care.

> *Most people see the doctor they want to see, so I think from a continuity point of view we know our patients very well and we've all been here a long time. [Group interview Lovell GP1]*

Arranging reviews

Administrative survey data from 15 intervention practices showed variation in the way practices arranged reviews (Table 2). Ten practices involved the whole administrative team, but in four, one or two administrators arranged 3D reviews in isolation. Reach was lowest in these four practices. In the remaining practice (Cabot), a dedicated research nurse arranged all the reviews, bypassing the administrative team. Notably, some 3D patients received the 3D reviews in addition to, rather than instead of their usual individual condition reviews, as intended.

> *I think there became a problem where patients were being invited in for their 3D and then a couple of months later, they'd get invited in for their diabetes and their asthma because one person up there wasn't talking to the other one. [Interview Blackwell Nurse 1]*

At Lovell and Harvey, existing arrangements for long-term condition reviews (one of the sampling criteria) underpinned the 3D review arrangements, reducing confusion. At Davy, the two administrators involved had to set up a different system for 3D patients. Being a large practice in which the rest of the administrative team were unaware of 3D requirements, difficulties arose when patients needed to re-arrange the appointment. At Beddoes, clinical and administrative staff decided collectively how they would implement the administrative aspects of 3D, but it differed from usual arrangements.

For peer review only - http://bmjopen.bmj.com/site/about/guidelines.xhtml

We'd had a team meeting after the training with the senior nurse and the GPs to decide what was the best way forward and then I met with the admin team to say, "What would you like to see on your screen so that you know they're part of the 3D study and so that you know about the appointments?" (Interview Beddoes practice manager)

Overall, adoption was inconsistent, affected by practices' choices in respect of continuity and arrangements for reviews. Duplication of reviews in some practices suggests difficulty in testing effectiveness of an intervention in a research situation that involves a short-term alteration to accustomed methods of providing care, that affects only a sub-set of patients.

Reach and maintenance

Table 3 shows mean reach in all intervention practices. We defined intervention reach in terms of receipt of planned 3D reviews by participating patients. Reach varied between practices from 38% and 94% (median 66%) of all recruited patients in a practice receiving both the nurse and GP appointments in first round reviews, and between 0% and 93% (median 47%) in second round reviews. Initial implementation of the intervention was therefore not well-maintained.

In the four initially-sampled practices, the qualitative data revealed contextual factors reducing the time window for delivering reviews. Lovell started delivering 3D reviews straight after training and had the highest reach of any practice in the intervention arm. The other three practices delayed starting, Davy because of the sudden loss of three of their long-term condition nurses and two GPs, Harvey because they were changing their system for sending letters re-calling patients for long-term reviews, and Beddoes because of staff sickness. Once started, Davy administrators struggled to organise reviews, hampered by ongoing sickness in the nursing team, and only managed to schedule 25% of the reviews required. The greatest challenge was accommodating paired reviews within over-stretched appointment schedules.

For peer review only - http://bmjopen.bmj.com/site/about/guidelines.xhtml

And I think because you're trying to tally it up with the doctor and the nurse, trying to find the time with the nurse if they've got more than one problem … and again they're not full time; they work part time. [Interview Davy Administrator 1]

Difficulties with arranging appointments reinforced practices' initial fears that the time demand and workload of implementing the 3D intervention would be too great. One suggestion made by GPs was that patients could be selected using more stringent criteria to reduce the overall number and maximise the chance of benefit. Another suggestion, from nurses, GPs and patients, was that the reviews need not involve the GP every time and/or could be shorter. Some comments suggested a lack of perceived value of the second-round reviews and that a second-round review with the nurse alone would be more time-efficient.

I know they need to be reviewed but do they need to be reviewed by nurse and GP? … because if we saw them for review and they were happy. Do they honestly need to see the GP to say "Are you still happy, like from last week"? [Interview Guppy Nurse 1]

Practices may therefore have been less motivated to arrange second reviews, and one practice reported that fewer patients responded to the invitation to attend them.

As a practice we've actually struggled to get them in for their second ones … we've written to them all twice – probably 30% of them haven't booked in and so we have had a bigger DNA rate for the second ones than the first ones. [Interview Beddoes GP1]

Overall, reach and maintenance were lower than intended, indicating a degree of implementation failure. Attention to context showed this was mainly a result of unanticipated events (e.g. staff loss or sickness) affecting practice capacity. However, aspects of intervention design (e.g. the inclusion of two reviews in one year with both nurse and GP each time) may also have impacted reach and maintenance.

For peer review only

For peer review only - http://bmjopen.bmj.com/site/about/guidelines.xhtml

Delivery of 3D review components

In 3D reviews that took place, each of the intervention components (see Figure 1) detected by the electronic search were completed in at least 92% of the delivered reviews, except medication adherence which was completed in 84% and printing the health plan in 77% (Table 4 and Appendix 4). The qualitative data provided insight into reasons for less consistently recorded components but also found evidence of significant variation in the manner of delivery suggesting that the high recorded component completion concealed some tick-box compliance. Variation in the patient-centredness of review component delivery has been reported in more detail in a previous paper [14]; here we focus primarily on implementation fidelity.

Eliciting and documenting the patient's concerns (most important problem noted)

The most consistently delivered component (99% completion) (Table 4), was asking patients about the health problems important to them. Nurses often invited disclosure of all health concerns, large or small.

> *She said to me, 'Is there anything you want to discuss with me at all, anything?' [Focus group Beddoes Patient 4]*

Some GPs and nurses commented on the value and novelty of asking about all patients' health concerns at the start of the consultation [14] but others were conscious of their clinical responsibility for managing the long-term conditions. Therefore, they preferred to separate the long-term conditions from health concerns they viewed as more trivial, or disabilities not amenable to change.

> *They want to discuss … the things that are happening to them at that particular moment … they've got a bad cold, or the cat's died or something else and they don't want to talk about their diabetes or their COPD. [Interview Beddoes GP3]*

There was also observed variation in how patient's concerns were elicited, recorded in the agenda and addressed in the health plan. The printed agenda was intended to reflect the patient's

For peer review only - http://bmjopen.bmj.com/site/about/guidelines.xhtml

perception of health problems (as well as clinical concerns), but nurses were often observed to reframe patients' problems into more medical terms. For example, one patient said: '*I can't take these naproxen now because … they've upset my stomach'* and the nurse recorded 'gastric problems'. This medicalisation of problems may have contributed to some patients' perception that the agenda was simply a means for the nurse to communicate their findings to the GP, rather than an agenda that the patient owned.

> *They just went through everything, all the problems, the nurse did and just wrote this report out for [GP2]. [Focus group Beddoes Patient 11]*

Quality of life and depression screening

Although completion was high, observation revealed that components that had a range of set answers were sometimes delivered in a 'tick-box' way that did not invite dialogue. This most commonly happened with template questions about quality of life and depression screening. It usually occurred when the nurse anticipated no problems being revealed but in interview some nurses also said that they lacked confidence in talking to patients about mental health.

Printing patient agenda

The patient agenda was printed in the vast majority of cases (93%) (Table 4) but problems with printing were occasionally observed and one nurse said she asked patients if they wanted it and that they declined. This may have reflected a perceived lack of ownership of the agenda by the patient.

> *Would you like a copy? And they're like, it's fine…Nobody has wanted a copy. [Interview Davy Nurse 1]*

Medication adherence

The completion rate of this component was lower at 84% but the qualitative data did not reveal why, other than some GPs' preference to complete the template after the review, which may have

For peer review only - http://bmjopen.bmj.com/site/about/guidelines.xhtml

meant they forgot to ask about it. On the contrary, there was evidence of some support for this component among GPs.

> *I do think the thing about tablets that patients take and which ones they don't like, if any, is useful. [Interview Lovell GP1]*

Collaboratively agreeing a plan

Health plans were intended as collaborative agreements between patient and GP, recording identified problems and specific actions for patient and GP to address each recorded problem. The patient and GP actions were well completed (93% and 92% respectively for the first problem) but the health plan was printed less frequently (77%) (Table 4). This may reflect GPs apparent dislike of the health plan and a perceived lack of value, as well as observed technical difficulties printing the plan. Interview data included reservations about the formulation of the health plan, which may have made GPs reluctant to give them to patients.

> *I felt it was almost that you were actually chiding them in some ways, to say, 'You should do this, should do that. … It's almost like when we were at primary school, taking home your homework tasks and goals for the week'. [Group interview Lovell GP3]*

During observations, a collaborative dialogue based on patients' chosen goals was seldom generated, and most plans were based on actions suggested by the GP. Some GPs commented that patients had not given prior thought to what they wished to address and that sometimes it was difficult to identify problems to include in the plan.

> *That's where I think perhaps them thinking in advance about their goal setting would help aid the conversation because often they say "No, no there's nothing I want to discuss" and you eventually tease out one or two things from them. [Interview Beddoes GP1]*

For peer review only - http://bmjopen.bmj.com/site/about/guidelines.xhtml

Some clinicians felt that the training provided by the trial team was insufficient to enhance skills required for agenda setting and especially collaborative action-planning.

> *I think some kind of communication training … would have been useful…there was a little bit about goal setting and confidence skills but there was no real practical element to it so in some ways you're testing what we already do but in a different context. [Interview Lovell GP1]*

Others would have liked some training follow-up to check if they were delivering the intervention as intended, and additional training prior to the second round of reviews to ensure they were '*doing it right*'.

In conclusion, although the quantitative data indicated that the intervention components were delivered for a high proportion of patients receiving reviews, the qualitative data showed that delivery style varied in ways that could sometimes compromise their function. Some components, such as creating the health plan, could have benefitted from more training.

Discussion

Summary of findings

The process evaluation identified that implementation was somewhat deficient in adoption (arranging the requisite number of 3D reviews, ensuring continuity of care, reducing the overall number of reviews) and aspects of delivery (creating health plans), but most delivered reviews included all components. Reasons for incomplete implementation included unexpected pressure on resources, implementation choices made by practices (including not involving the entire administrative team), and insufficient training for using patient-centred approaches. During delivery of reviews to patients, using the template was the key to maintaining 'fidelity of form', but variation in the patient-centredness of delivery sometimes undermined 'fidelity of function' [28]. The overall prediction made by the process evaluation team while blind to the trial results was that the

For peer review only

For peer review only - http://bmjopen.bmj.com/site/about/guidelines.xhtml

intervention would have improved patient experience in patients who received 3D reviews, but not changed health-related quality of life (the findings were presented and this prediction made at the Trial Steering Committee meeting immediately before unblinding). The prediction of improved experience was based on the positive feedback from patients in focus groups and interviews suggesting improvements in their perceptions of care. The prediction of unchanged health-related quality of life was based on limited engagement of patients in the health plans (observed and described by clinicians), a lack of evidence of major changes to quality of care and feedback from administrators and clinicians about difficulties organising reviews. The trial results confirmed these predictions [12], which increases our confidence in the process evaluation findings.

Strengths and weaknesses

Strengths include pre-designing the process evaluation based on a published framework for process evaluation of cluster-randomised trials [10, 17, 18] covering all trial stages, and maintaining responsiveness to emerging information. This maximised the likelihood that all factors that might influence implementation fidelity, including context, were considered [7]. Data of varying and complementary types were collected from a wide range of sources, both purposively sampled and cross-trial. The purposive sampling of practices mitigated the limitation that only a subset of practices and individuals involved in the trial were interviewed or observed, and we explored the full range of variation in implementation and reach (Table 2), including quantitative process data from all practices. In accordance with published guidance [10], the process evaluation analysis took place blind to the trial results.

Comparison to other literature

An aim of the 3D process evaluation was to examine implementation fidelity to distinguish between implementation failure and intervention failure in the event of a null result. This distinction matters because it is important to avoid discarding a potentially effective intervention that was poorly implemented [10, 29, 30]. Implementation difficulties and deficiencies are not infrequently

For peer review only - http://bmjopen.bmj.com/site/about/guidelines.xhtml

identified in effectiveness evaluations of complex health care delivery interventions [31-34] but are not always elucidated [20, 35]. In this study we found evidence of a degree of implementation failure and, in addition to identifying poorly implemented components, we have considered reasons for poor implementation and whether they are modifiable. Non-modifiable reasons include unexpected events in individual practices, most commonly staff leaving and not being easily replaceable. Potentially modifiable reasons for adoption problems include the individual choices practices made about arranging reviews, influenced by practice size and existing recall systems, but implementation was also affected by the research trial context. Implementation in these circumstances is short-term, and only applies to a sub-set of patients, with the majority still receiving usual care, which increases the risk of confusion and duplication. This circumstance influenced administrative choices made by practices, which in turn affected implementation.

The role of intervention design and set-up, including training provided by research teams to practices, is significant and modifiable. In common with other research teams, we experienced difficulty in establishing a new way of working [36, 37], although care *did* change enough that patients reported statistically significant changes in their experience of care in the intended direction (e.g. having a greater sense of being consulted about their experience of health) and greater satisfaction with their care [12]. The evidence suggested that this was attributable to the design of the intervention reviews (longer, comprehensive, and asking first about the patient's concerns) [14], but there was also evidence that intervention design negatively affected implementation in some potentially modifiable ways. Implementation of health plans suffered from insufficient training and a lack of coherence between the health plan format and GP current practice, clearly suggesting that intervention design relating to both these aspects could be improved. Professional perceptions that some patients were unprepared to engage in health planning suggests that additional patient-targeted intervention components and/or better clinician training addressing attitudes and barriers to engaging in health-planning and supporting self-

For peer review only - http://bmjopen.bmj.com/site/about/guidelines.xhtml

management [38] might facilitate collaboratively agreeing a plan of action. Many professionals did not see value for many patients in doing a second comprehensive review in the same year, which likely contributed to lower reach for second reviews, and suggests that more targeted follow-up might have been a better design than routine re-review for all.

Our overall judgement was that there was therefore evidence of both implementation failure and intervention failure, but that these were linked rather than truly distinct because in this case aspects of intervention design influenced implementation. Improvements in intervention design could be focused on incorporating skills practice in the 3D training, better selection and preparation of patients, improvement to the health plan including a different format and greater patient ownership. We could also consider greater flexibility in follow-up reviews to allow varying intensity of follow-up tailored to patient need.

There is however a dilemma between ensuring an intervention is implemented with high fidelity and allowing flexibility to suit local circumstances. The intervention design did allow for some adaptation ‘at the periphery’ [39] and distinguished between core components that must be implemented in a particular form and less closely specified components whose form could vary, as long as the intended function was achieved [28]. This is recommended to facilitate implementation in individual practices, but it is not straightforward to choose where to specify intervention elements as ‘central’ and where to allow flexibility. In retrospect, some flexibility in follow-up reviews would be reasonable in future iterations of this type of intervention. A further change which might plausibly alter impact on health-related quality of life would be to evaluate implementation over a longer period (although that clearly has significant cost implications) or as a whole practice improvement intervention delivered to all eligible patients, rather than running a parallel system of care for individual trial participants. However, this creates the paradox that providing an intervention outside the context of a research trial may be more likely to provide a true representation of its effectiveness, but the effectiveness cannot be proved without the research.

For peer review only

Conclusions

In the context of an intervention that followed the recommendations and best evidence for care of people with multimorbidity, where the trial provided strong evidence that there was no effect on the primary outcome of HRQoL but an improvement in patient-centred outcomes, we found evidence of both implementation and intervention failure. Although this challenges the assumption that implementation and intervention failure can be clearly distinguished, we believe that the distinction does provide a useful framework to help interpret trial findings and to systematically identify modifiable and non-modifiable factors to inform future implementation decisions. This paper provides a worked example of how to use these concepts in process evaluation. We conclude firstly, that in the case of the 3D trial a truer test of the intervention effectiveness might be achieved by modifications that support better implementation, including whole practice implementation over a longer period to allow embedding. Secondly, it is important to examine reasons for implementation deficiencies to determine not only whether there were implementation failures but also the reasons for them and whether they might be modifiable in order to avoid discarding a potentially effective intervention.

Ethics approval

The trial and process evaluation were approved by the South-West England NHS Research Ethics Committee (14/SW/0011)

Consent for publication

Not applicable as all data have been anonymised data

For peer review only - http://bmjopen.bmj.com/site/about/guidelines.xhtml

Availability of data and materials

All data relevant to the study are included within the article or uploaded as supplementary information. Additional qualitative data are available from the corresponding author on reasonable request.

Competing interests

The authors declare that they have no competing interests

Funding

This project was funded by the National Institute for Health Research Health Services and Delivery Research Programme (project number 12/130/15). The views expressed are those of the author(s) and not necessarily those of the NIHR or the Department of Health and Social Care.

Authors contributions:

CM, AS and BG designed the process evaluation. CS led the design of the 3D intervention and the randomised trial. CM collected and analysed the qualitative data with input from AS, LW and BG and led the analysis and write-up of the results presented in this paper. AS, BG and CS critically revised the manuscript. KC helped to design the template, analysed the quantitative data it recorded and helped to collect administrative survey data. M-SM contributed to the design of the process evaluation and facilitated data collection in the role of trial manager. All authors discussed findings, commented on the paper and approved the final version.

Acknowledgements:

The authors sincerely thank all those practices, staff and patients who took part in the 3D trial and especially those who contributed to the process evaluation by consenting to interviews and

For peer review only - http://bmjopen.bmj.com/site/about/guidelines.xhtml

observation. We thank Sue Cooke and Polly Duncan who collected and helped with the analysis of some of the qualitative data in the lead trial site, Rebecca Robinson who supported the process evaluation with data collection in the early stages, Daisy Gaunt who did the main quantitative analysis for the trial, and the researchers in supporting trial sites, Bridie FitzPatrick, John McLeod, Caroline Gardner, Victoria Lee and Keith Moffat who supported the process evaluation by facilitating data collection and collecting administrative survey data. We also thank Pete Bower and Stewart Mercer, the Principle Investigators at those sites, who contributed to trial team discussions on the process evaluation design and findings. We also acknowledge other members of the wider trial team, Sandra Hollinghurst, Bryar Kadir, Emma Moody, Imran Rafi, and Joanna Thorn. Finally, we thank the Patient Public Involvement group who contributed valuable insight into qualitative findings and great enthusiasm for the study.

For peer review only

For peer review only - http://bmjopen.bmj.com/site/about/guidelines.xhtml

Table 1: Data from intervention practices used for this study

Data	Sampled intervention practices.	Data sources:	Data used to examine:
Electronic data capture	All	3D electronic template recording of reviews completed and review components delivered to all patients	Reach and maintenance<br>Fidelity of delivery of intervention components to patients
Administrative survey	All	Research team completed questionnaire about organisation of reviews in all intervention practices	Adoption, reach and maintenance
Baseline interviews	Beddoes, Davy, Harvey, Lovell	4 administrators, 4 nurses, 5 GPs	Individual practice context to understand adoption and reach.
3D review observations	Beddoes, Davy, Harvey, Lovell, Cabot, McReady, Guppy, Carpenter	13 nurses, 15 GPs, 22 patients[1]	Variation in delivery of intervention components to patients
Post review debriefs and informal interviews	Beddoes, Davy, Harvey, Lovell, Cabot, McReady, Guppy, Carpenter	12 nurses, 7 GPs, 10 patients	Variation in delivery of intervention components to patients<br>Maintenance of intervention delivery
Patient focus groups	Beddoes, Davy, Harvey, Lovell	22 patients[2]	Variation in delivery of intervention components to patients
End-of trial interviews	Beddoes, Davy, Harvey, Lovell, Blackwell	4 administrators, 6 nurses, 5 GPs, 7 patients	Variation in delivery of intervention components to patients.<br>Maintenance of intervention delivery

1. 6 patients were observed for both parts of review
2. 2 focus groups of 3 patients, 1 focus group of 7 patients and 1 focus group of 7 patients and 2 carers

For peer review only

For peer review only - http://bmjopen.bmj.com/site/about/guidelines.xhtml

Table 2: Intervention practices

Practice	Practice size	Combined reviews at baseline[1]	Admin involvement	3D review organisation[3]	Reach	Qualitative data collection[4]
Lovell	4,000 patients<br>4 GPs, 2 nurses	All combined	1 administrator. All aware	Appointment sent, review appointments paired	First review 94%<br>Second review 93%	In depth. All elements
Tothill	10,000 patients<br>40 GPs, 4 nurses	Some combined	All	Appointment sent, review appointments separate	First review 92%<br>Second review 86%	None
Macready	6,000 patients<br>6 GPs, 2 nurses	All combined	1 administrator. All aware	Appointment sent, review appointments paired	First review 92%<br>Second review 50%	Observation and post-review informal interview
Dunbar	15,000 patients<br>16 GPs, 5 nurses	All combined	All	Letter inviting patient to call, review appointments paired	First review 90%<br>Second review 75%	None
Cabot	10,000 patients<br>12 GPs, 5 nurses	Some combined	Research nurse only	Appointment sent, review appointments separate	First review 83%<br>Second review 74%	Observation and post-review informal interview
Beddoes	5,500 patients,<br>4 GPs, 3 nurses	All combined	All	Letter inviting patient to call, review appointments separate	First review 80%<br>Second review 82%	In depth. All elements
Guppy	8,000 patients<br>6 GPs, 3 nurses	All combined	1 administrator. All aware	Appointment sent, review appointments paired	First review 80%<br>Second review 76%	Observation and post-review informal interview
Penn	10,500 patients<br>9 GPs, 3 nurses	Some combined	1 administrator. All aware	Phone call to patient, review appointments paired	First review 80%<br>Second review 47%	None
Harvey	15,000 patients<br>13 GPs, 4 nurses	Some combined	All	Appointment sent, review appointments sometimes separate	First review 77%<br>Second review 44%	In depth All elements
Priestman	13,500 patients<br>10 GPs, 3 nurses	All combined	All	Letter inviting patient to call, review appointments paired	First review 75%<br>Second review 45%	None
Sharples	4,500 patients<br>4 GPs, 2 nurses	None combined	All	Letter inviting patient to call, review appointments separate	First review 71%<br>Second review 67%	None
Martineau	5,000 patients<br>4 GPs, 2 nurses	Some combined	2 administrators. Others unaware	Phone call to patient, review appointments paired	First review 69%<br>Second review 53%	None
Carpenter	14,500 patients<br>12 GPs, 4 nurses	All combined	Unsure if all aware	Letter inviting patient to call, review appointments paired	First review 67%<br>Second review 50%	Observation and post-review informal interview
Blackwell	13,500 patients<br>9 GPs, 7 nurses	All combined	Nurse and administrator. Others unaware.	Letter inviting patient to call, nurse completed both parts of review	First review 66%<br>Second review 9%	End of trial interviews
Davy	14,500 patients<br>12 GPs 5 nurses	Some combined	2 administrators. Others unaware	Appointment sent, later review appointments separate	First review 38%<br>Second review 0%	In depth. All elements

(1) Combined reviews means reviews were purposely arranged to include all long-term conditions where there was a nurse-led clinic. (2) Continuity of care based on visit entropy score; lower scores indicate greater continuity: High<50, Medium 50-60, Low>60. (3) Paired means that nurse and GP appointments made at the same time but could take place on different days. (4) See table 1 for details of qualitative data collected.

For peer review only - http://bmjopen.bmj.com/site/about/guidelines.xhtml

Table 3: Quantitative evaluation of reach

	No (%) of 3D reviews delivered
Practice level analysis	N= 16 practices
Reach (% expected number of reviews delivered)	
First review	Median 66% (range 38-94%)
Second review	Median 47% (range 0-93%)
Patient level analysis	N= 797
Delivery of 3D nurse and GP reviews[a]	
Two 3D reviews with both GP and nurse (full)	390 (49%)
One 3D review with both GP and nurse (partial)	205 (26%)
Other (eg nurse review but no GP review) (partial)	31 (4%)
No 3D reviews (none)	171 (21%)

[a] 622 (78%) patients had at least one nurse review; 599 (75%) had at least one GP review. 390 (49%) patients received a 'full' intervention (defined as having two reviews, with each review involving a nurse and a GP appointment which could be on the same day or different days i.e. four appointments in total) in the 15 months of follow-up. 21% received no intervention.

Table 4: Quantitative evaluation of component delivery

	No (%) of each element of the 3D review delivered
Delivery of pharmacist medication review	607/797 (76%)
For those with at least one GP or nurse review	
Most important problem noted (patient agenda)[1]	616/622 (99%)
EQ5D pain question noted (Quality of life)[1]	611/622 (98%)
PHQ9 depression screening noted[1]	599/622 (96%)
Patient agenda printed[1]	579/622 (93%)
Medication adherence noted[2]	506/599 (84%)
First patient problem noted[2]	590/599 (98%)
Noted 'what patient can do' for first problem (health plan)[2]	559/599 (93%)
Noted 'what GP can do' for first problem (health plan)[2]	554/599 (92%)
3D health plan printed[2]	461/599 (77%)

[1] Components delivered in the nurse part of the review of which 622 took place. If one patient had two reviews, this component was delivered in at least one

[2] Components delivered in the GP part of the review of which 599 took place. If one patient had two reviews, this component was delivered in at least one

For peer review only - http://bmjopen.bmj.com/site/about/guidelines.xhtml

References

1. American Geriatrics Society Expert Panel on the Care of Older Adults with Multimorbidity. Patient-centered care for older adults with multiple chronic conditions: a stepwise approach from the American Geriatrics Society: American Geriatrics Society Expert Panel on the Care of Older Adults with Multimorbidity. J Am Geriatr Soc. 2012;60(10):1957-68.
2. Muth C, van den Akker M, Blom JW, Mallen CD, Rochon J, Schellevis FG, et al. The Ariadne principles: how to handle multimorbidity in primary care consultations. Bmc Medicine. 2014;12.
3. National Institute for Health and Care Excellence. Multimorbidity: clinical assessment and management (NG56) [PDF]. London UK: National Institute for Health and Care Excellence (NICE); 2016 [Available from: https://www.nice.org.uk/guidance/ng56].
4. Man MS, Chaplin K, Mann C, Bower P, Brookes S, Fitzpatrick B, et al. Improving the management of multimorbidity in general practice: protocol of a cluster randomised controlled trial (The 3D Study). BMJ Open. 2016;6(4):e011261.
5. O'Cathain A, Thomas KJ, Drabble SJ, Rudolph A, Hewison J. What can qualitative research do for randomised controlled trials? A systematic mapping review. BMJ Open. 2013;3(6).
6. Oakley A, Strange V, Bonell C, Allen E, Stephenson J, Team RS. Process evaluation in randomised controlled trials of complex interventions. BMJ. 2006;332(7538):413-6.
7. Carroll C, Patterson M, Wood S, Booth A, Rick J, Balain S. A conceptual framework for implementation fidelity. Implement Sci. 2007;2:40.
8. Rychetnik L, Frommer M, Hawe P, Shiell A. Criteria for evaluating evidence on public health interventions. J Epidemiol Community Health. 2002;56(2):119-27.
9. Greenhalgh T, Papoutsi C. Studying complexity in health services research: desperately seeking an overdue paradigm shift. BMC Med. 2018;16(1):95.
10. Moore G, Audrey S, Barker M, Bond L, Bonell C, Hardeman W, et al. Process evaluation of complex interventions. UK Medical Research Council (MRC) guidance. MRC Population Health Science Research Network; 2014 2014.
11. Chaplin K, Bower P, Man MS, Brookes ST, Gaunt D, Guthrie B, et al. Understanding usual care for patients with multimorbidity: baseline data from a cluster-randomised trial of the 3D intervention in primary care. BMJ Open. 2018;8(8):e019845.
12. Salisbury C, Man M-S, Bower P, Guthrie B, Chaplin K, Gaunt DM, et al. Management of multimorbidity using a patient-centred care model: a pragmatic cluster-randomised trial of the 3D approach. The Lancet. 2018;392(10141):41-50.
13. Salisbury C, Man MS, Chaplin K, Mann C, Bower P, Brookes S, et al. A patient-centred intervention to improve the management of multimorbidity in general practice: the 3D RCT. Health Services and Delivery Research: NIHR. 2019;7.
14. Mann C, Shaw A, Wye L, Salisbury C, Guthrie B. A computer template to enhance patient-centredness in multimorbidity reviews: a qualitative evaluation in primary care. Br J Gen Pract. 2018;68(672):e495-e504.
15. Hoffmann TC, Glasziou PP, Boutron I, Milne R, Perera R, Moher D, et al. Better reporting of interventions: template for intervention description and replication (TIDieR) checklist and guide. BMJ. 2014;348:g1687.

For peer review only - http://bmjopen.bmj.com/site/about/guidelines.xhtml

16. Mann C, Chilcott S, Plumb K, Brooks E, Man MS. Reporting and appraising the context, process and impact of PPI on contributors, researchers and the trial during a randomised controlled trial - the 3D study. Res Involv Engagem. 2018;4:15.
17. Mann C, Shaw A, Guthrie B, Wye L, Man MS, Hollinghurst S, et al. Protocol for a process evaluation of a cluster randomised controlled trial to improve management of multimorbidity in general practice: the 3D study. BMJ Open. 2016;6(5):e011260.
18. Grant A, Treweek S, Dreischulte T, Foy R, Guthrie B. Process evaluations for cluster-randomised trials of complex interventions: a proposed framework for design and reporting. Trials. 2013;14:15.
19. Malterud K, Siersma VD, Guassora AD. Sample Size in Qualitative Interview Studies: Guided by Information Power. Qual Health Res. 2015;26(13):1753 –60.
20. Wells M, Williams B, Treweek S, Coyle J, Taylor J. Intervention description is not enough: evidence from an in-depth multiple case study on the untold role and impact of context in randomised controlled trials of seven complex interventions. Trials. 2012;13:95.
21. Ovretveit J. Understanding the conditions for improvement: research to discover which context influences affect improvement success. BMJ Qual Saf. 2011;20 Suppl 1:i18-23.
22. May CR, Mair FS, Dowrick CF, Finch TL. Process evaluation for complex interventions in primary care: understanding trials using the normalization process model. BMC Fam Pract. 2007;8:42.
23. Murray E, Treweek S, Pope C, MacFarlane A, Ballini L, Dowrick C, et al. Normalisation process theory: a framework for developing, evaluating and implementing complex interventions. BMC Med. 2010;8:63.
24. Tong A, Sainsbury P, Craig J. Consolidated criteria for reporting qualitative research (COREQ): a 32-item checklist for interviews and focus groups. Int J Qual Health Care. 2007;19(6):349-57.
25. Sandelowski M. Whatever happened to qualitative description? Res Nurs Health. 2000;23(4):334-40.
26. Braun V, Clarke V. Using thematic analysis in psychology. Qualitative research in psychology. 2006;3(2).
27. Bice TW, Boxerman SB. A quantitative measure of continuity of care. Med Care. 1977;15(4):347-9.
28. Hawe P, Shiell A, Riley T. Complex interventions: how "out of control" can a randomised controlled trial be? BMJ. 2004;328(7455):1561-3.
29. Basch CE, Sliepcevich EM, Gold RS, Duncan DF, Kolbe LJ. Avoiding type III errors in health education program evaluations: a case study. Health Educ Q. 1985;12(4):315-31.
30. Campbell NC. Designing and evaluating complex interventions to improve healthcare. BMJ. 2007;334:455-9.
31. Kennedy A, Rogers A, Chew-Graham C, Blakeman T, Bowen R, Gardner C, et al. Implementation of a self-management support approach (WISE) across a health system: a process evaluation explaining what did and did not work for organisations, clinicians and patients. Implement Sci. 2014;9:129.
32. Bamford C, Poole M, Brittain K, Chew-Graham C, Fox C, Iliffe S, et al. Understanding the challenges to implementing case management for people with dementia in primary care in England: a qualitative study using Normalization Process Theory. BMC Health Serv Res. 2014;14:549.

For peer review only - http://bmjopen.bmj.com/site/about/guidelines.xhtml

33. Grant A, Dreischulte T, Guthrie B. Process evaluation of the Data-driven Quality Improvement in Primary Care (DQIP) trial: case study evaluation of adoption and maintenance of a complex intervention to reduce high-risk primary care prescribing. BMJ Open. 2017;7(3):e015281.
34. Jansen YJ, de Bont A, Foets M, Bruijnzeels M, Bal R. Tailoring intervention procedures to routine primary health care practice; an ethnographic process evaluation. BMC Health Serv Res. 2007;7:125.
35. Bunce AE, Gold R, Davis JV, McMullen CK, Jaworski V, Mercer M, et al. Ethnographic process evaluation in primary care: explaining the complexity of implementation. BMC Health Serv Res. 2014;14(607):607.
36. Kennedy A, Chew-Graham C, Blakeman T, Bowen A, Gardner C, Protheroe J, et al. Delivering the WISE (Whole Systems Informing Self-Management Engagement) training package in primary care: learning from formative evaluation. Implement Sci. 2010;5(1):7.
37. Kennedy A, Rogers A, Bowen R, Lee V, Blakeman T, Gardner C, et al. Implementing, embedding and integrating self-management support tools for people with long-term conditions in primary care nursing: a qualitative study. Int J Nurs Stud. 2014;51(8):1103-13.
38. Eaton S, Roberts S, Turner B. Delivering person centred care in long term conditions. BMJ. 2015;350:h181.
39. Damschroder LJ, Aron DC, Keith RE, Kirsh SR, Alexander JA, Lowery JC. Fostering implementation of health services research findings into practice: a consolidated framework for advancing implementation science. Implement Sci. 2009;4:50.

For peer review only - http://bmjopen.bmj.com/site/about/guidelines.xhtml

Figure 1: 3D intended intervention work and core components

Adoption by the practice – intended administrative activity

- Identify patients with $\geqslant$ 3 long-term conditions and flag on EMIS
- Install purpose-designed electronic 3D review template
- In consultation with clinicians, allocate a named GP (and nurse if appropriate) for all reviews
- All appointments outside reviews scheduled with named GP and/or nurse and offered as longer appointments
- Schedule participating patients for 6 monthly 3D review of all conditions together in extended two-part appointments, first part with named nurse, second part with named GP
- Cancel usual long-term condition reviews, and replace with 3D review
- Run monthly monitoring searches and send them to researchers

Core components

- Continuity of care
- A comprehensive review arranged with named nurse and GP in separate appointments every six months
- Longer appointments with named GP or nurse as needed between reviews

3D multimorbidity reviews – intended GP, nurse and pharmacist activity

- Two-part long-term condition review with named nurse and GP, to address all conditions together, using new 'intelligent' 3D review template.
- Part 1 typically done by a nurse: identify patient's priorities and quality of life issues, screen for depression and complete disease checks. Create agenda for second part of review based on this information and give printed copy to patient.
- Pharmacist review of medication prior to part 2
- Part 2 typically done by a GP: address agenda, review treatment and medication adherence, aim to optimise medication and reduce treatment burden, agree health plan with patient and provide written copy
- Involvement of secondary care physician if needed

Core components

- Compile patient agenda based on patient priorities and clinical measures and provide copy to patient
- Depression screening
- Attention to quality of life
- Chronic disease monitoring
- Medication review and adherence
- Share printed health plan with actions for both patient and GP

For peer review only

Figure 2: Process evaluation design and research questions (research stages addressed in this paper are shown in blue)

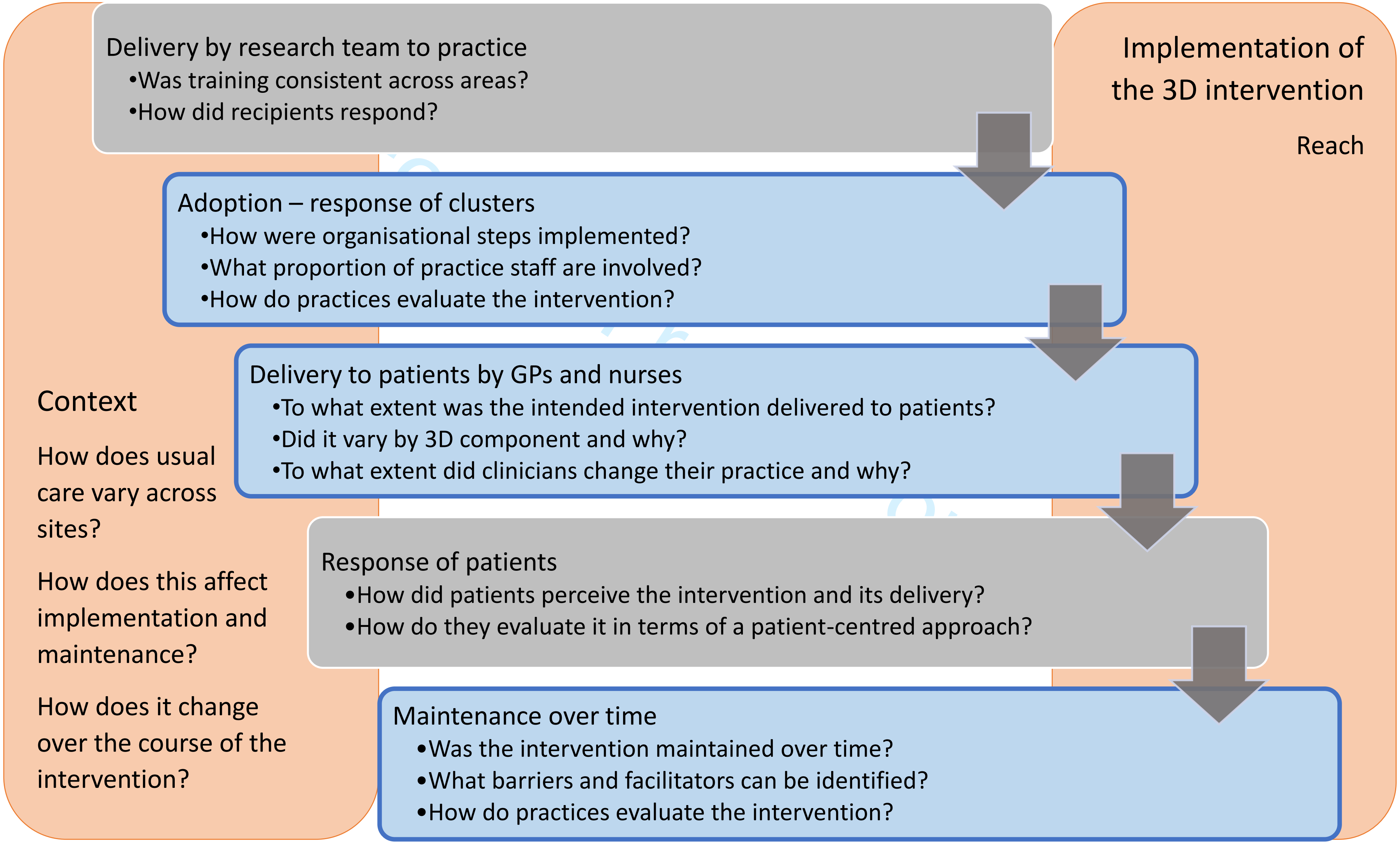

Appendix 1: Tidier checklist for the 3D intervention

Additional information can be found in the published full report of the trial: Salisbury C, Man M-S, Chaplin K, Mann C, Bower P, Brookes S, et al. A patient-centred intervention to improve the management of multimorbidity in general practice: the 3D RCT. Health Serv Deliv Res 2019;7(5)

Item No	Item		Summary information and location of full detail in report
**Brief name**			
1	Provide the name or a phrase that describes the intervention	✔	Improving the management of multimorbidity in general practice – the 3D study
**Why**			
2	Describe any rationale, theory, or goal of the elements essential to the intervention	✔	Underlying theoretical basis is the Patient-centred Care Model. Intervention designed to address problems experienced by people with multimorbidity and aimed to achieve improved quality of life.<br>*Report Pages 3, 9*
**What**			
3	Materials: Describe any physical or informational materials used in the intervention, including those provided to participants or used in intervention delivery or in training of intervention providers. Provide information on where the materials can be accessed (such as online appendix, URL)	✔	An purpose-designed IT template was used within Egton Medical Information Systems (EMIS) which when completed generated a patient agenda and a patient health plan. Intervention patients received a 3D card which identified them to practices and specified their named GP.<br>*Report Pages 11-15 and Appendices 3, 5-8*<br>*Report Supplementary Material 1 and 2*
4	Procedures: Describe each of the procedures, activities, and/or processes used in the intervention, including any enabling or support activities	✔	This was highly complex intervention that incorporated:<br>Installing the EMIS template<br>Identifying and recruiting the target group<br>Allocating a named GP and nurse for each participant and issuing a 3D card to each participant to improve continuity of care.<br>Training the practice staff and clinicians<br>Organising and delivering 6 monthly 3D comprehensive reviews of all health conditions and of psychosocial factors that were delivered in 2 parts, first with the named nurse, second with the named GP.<br>Medication review by pharmacist viewing patient record remotely<br>Meetings of practice champions<br>Provision of monthly monitoring feedback to practices about their delivery of the intervention<br>*Report pages 10 -15*

For peer review only

Item No	Item		Summary information and location of full detail in report
**Who provided**			
5	For each category of intervention provider (such as psychologist, nursing assistant), describe their expertise, background, and any specific training given	✔	Intervention providers included GPs, nurses in general practice, pharmacists, general practice administrators and receptionists, and one secondary care physician for each area.<br>*Report page 12*
**How**			
6	Describe the modes of delivery (such as face to face or by some other mechanism, such as internet or telephone) of the intervention and whether it was provided individually or in a group	✔	Face-to-face delivery of comprehensive 6 monthly reviews. Remote performance of medication review element<br>*Report pages 11-15*
**Where**			
7	Describe the type(s) of location(s) where the intervention occurred, including any necessary infrastructure or relevant features	✔	The intervention occurred in individual general practices in three areas of the UK
**When and How Much**			
8	Describe the number of times the intervention was delivered and over what period of time including the number of sessions, their schedule, and their duration, intensity, or dose	✔	The intervention two-part reviews were delivered twice in 12 months. The intervention components were mainly delivered in these reviews carried out in nurse appointments of 30-50 minutes and in GP appointments of 20<br>*Report pages 12-13*
**Tailoring**			
9	If the intervention was planned to be personalised, titrated or adapted, then describe what, why, when, and how	✔	Practices were allowed some flexibility in how intervention delivery was organised<br>*Report page 14*
**Modifications**			
10	If the intervention was modified during the course of the study, describe the changes (what, why, when, and how)		The intervention was modified after piloting from a whole practice service change intervention to selected patients only.<br>*Report page 16 and Appendix 14*
**How well**			
11	Planned: If intervention adherence or fidelity was assessed, describe how and by whom, and if any strategies were used to maintain or improve fidelity, describe them	✔	Mixed methods were used involving both quantitative and qualitative researchers in the trial team. Quantitative methods involved electronic monitoring of delivery of intervention components. Qualitative

For peer review only

Item No	Item		Summary information and location of full detail in report
			methods included interviewing participants and providers and observing delivery. Strategies to maintain and improve fidelity were the monthly electronic monitoring feedback, meetings of practice champions and financial incentives<br>*Report pages 31-33*
12	Actual: If intervention adherence or fidelity was assessed, describe the extent to which the intervention was delivered as planned	✔	Half the participants received the full intended number of reviews. In delivered reviews most components were delivered but the way they were delivered varied. This is presented and discussed in the conclusion of the present paper.<br>*Report pages 77-86*

For peer review only

Appendix 2: Interview topic guides and observation checklist

Topic guide for lead administrator pre-implementation

What do you think of the 3D intervention?

Please can you explain how you are organising the appointments and recall for 3D

How is it similar to what you already do or plan to do?

How is it different from what you already do or plan to do?

What did you think of the information you have been given?

How might it have been improved?

How do you think it might affect the practice?

Difficulties - What will be the main challenges?

Benefits

Roles of doctors, nurses and reception staff

How do you think it might affect your work?

Difficulties – what concerns do you have?

Benefits

How do you think it might change the patients' experience?

How might it affect different types of patient?

Is there anything else you would like to say?

Topic guide for lead nurse or lead GP pre-implementation

What do you think of the 3D intervention?

How is it similar to what you already do or plan to do?

How is it different from what you already do or plan to do?

What did you think of the training for 3D?

How might it have been improved?

How do you think it might affect your practice?

Difficulties - What will be the main challenges?

Benefits

Roles of doctors, nurses and reception staff

How do you think it might affect your work?

For peer review only

Difficulties – what concerns do you have?

Benefits

How do you think it might change the patients' experience?

How might it affect different types of patient?

Is there anything else you would like to say?

Topic guide for follow-on interviews after consultation observation – GP or nurse

How do you feel the consultation went?

What, if anything, would you have done differently and if so why?

What went particularly well?

How did the timing go?

How happy were you with how it was structured?

How easy was it to integrate use of the template?

How easy was it to get a complete picture of the patient's concerns?

How do you feel the patient responded?

What had you planned to talk about and/or what did you want to agree a plan for?

Was anything not covered that you had wanted to talk about?

GP only

How much did you use the nurse's agenda and how helpful was it?

How helpful was the medication review?

Were you happy with the plan? Do you think the patient was happy with the plan?

Was there anything that surprised you?

Topic guide for follow-on interviews after consultation observation – patients

How do you feel the consultation went?

What went particularly well or what did you particularly like?

What, if anything, were you not happy about? *(template, timing, any particular questions)*

How well do you feel the nurse understood what you were concerned about?

How well do you feel the doctor understood what you were concerned about?

What had you planned to talk about and what did you want to agree a plan for before you went into the appointment?

Was anything not covered that you had wanted to talk about?

For peer review only

What do you think you will discuss with the doctor? or What plan have you come away with?

Was there anything that surprised you?

How happy were you with the amount of time you had?

How conscious were you of the computer? Did it interfere with the discussion with the doctor/nurse

Topic guide for administrator post-implementation

What is your opinion of the intervention?

What perceived benefits, downsides, and unintended consequences both positive and negative?

How has it affected the management of LTCs?

How do you think patients have responded? Which patients do you think have benefitted most?

How difficult has it been to arrange appointments and to manage the searches etc?

What has your process been?

What helped the process?

What would have made it easier?

What elements of 3D do you think would be worth continuing?

Topic guide for GPs or nurses post-implementation

1. Response to intervention:

What has it been like taking part in the intervention?

How has it changed your practice if at all?

What perceived benefits, downsides, and unintended consequences both positive and negative?

How has it affected the roles of the nurse and doctor and team working in general? How has it affected the management of LTCs? (*Goal setting?)*

How do you think this intervention and your role in it supports patient-centred care, if at all?

How do you think patients have responded? Who do you think has benefitted most/least?

What difficulties have there been in delivering the intervention? *(How easy was it to organise their care in this way?)*

What helped to deliver the intervention? *(whole system change or pockets?)*

How adequate was the preparation by, and support from, the research team?

How you were able to integrate the template into your consultation or not i.e. did you use it? We realise it is not ideal for everyone and would like to know how it could be improved?

Are there any elements of the intervention that are particularly useful or need changing?

Identifying concerns

For peer review only

Depression screening
Goal negotiation
Care plans
Length of appointment
Pharmacy review
Continuity of care

2. Have your views on the intervention changed in any way from when it was first introduced?

3. Specific questions to follow up on early interview or on observed consultation

4. Maintenance:

What would encourage you to keep this system of care for multi-morbid patients?

What will you do now? Are there any elements you might take forward? If so why and if not why not? *(Distinguish between concept not being enough of a priority (if so why not?) and whether or not this is the right way to do it)*

Does anything need to change? What would make it easier to implement? What would you do differently?

How have local circumstances affected what you did? Has that changed during the study?

5. Is there anything else you would like to say?

Topic guide for patients – post intervention .

Focus group or individual interviews

Can you comment on the care you receive from your GP practice in general and for your long-term conditions in particular?

What is most important to you about the way your care is provided?

Is there anything that you would like to change/improve? If so how?

What do you think of the 3D system?

What, if anything, is different about your care?

Has it had any effect on your health?

Have you had any care or intervention that you don't think you would have had without 3D?

Would you like to see the 3D system continuing?

If it was not all continued what would be the most important parts to continue?

Consultation observation guide

Patient identification code:

Clinician: **GP practice:**

Conditions reviewed:

3D review part 1 or part 2:

Length of consultation: scheduled: actual:

For each consultation note:

- General appearance and demeanour of clinician and patient
- Physical set-up of the room e.g. location of computer in relation to clinician/patient (diagram)
- How the consultation is opened
- Whether/how the clinician talks about 3D and how it may impact organisation of the patient's care
- Actions taken by clinician since last appointment and the responses of patient/carer
- Actions taken by the patient since last appointment and the responses of the clinician
- How the 3D template is used and talked about by the clinician during the consultation
    - *e.g. does the clinician refer to it or use it as justification for certain questions? Does it impact clinician/patient verbal/non-verbal communication? Are there any technical problems with use of the template?*
- How the patient/carer appears to respond to use of the template during the consultation
    - *e.g. any comments made by the patient or questions asked about use of template. Does the patient welcome the provision of written agenda/care plan or not?*
- Whether/how the clinicians seeks to elicit the patient's concerns and priorities
- Was everything covered i.e. was it truly holistic and was everything that might affect health and wellbeing considered?
- What the clinician tells the patient about their condition(s) and the responses of patient/carer
- Information and knowledge exchange: Were appropriate questions asked by both patient and doctor and were the answers adequate and did the doctor check understanding?
- How medication adherence is discussed and medications reviewed
- How depression is discussed
- How treatment/care plans are talked about and negotiated – are goals set?

- Was there evidence at the end of agreement as to what needed to be addressed and how that would be done?
- Nature of the clinician/patient relationship and decision making during the consultation
    - *e.g. examples of patient-centredness, who is managing the consultation agenda, involvement of patient/carer in care and treatment planning, clinician respect for patient's values/preferences, checking understanding*
- Interaction (verbal and non-verbal) between clinician and patient/carer during the consultation
    - *e.g. how questions are asked, responses to questions, verbal/non-verbal cues, clinician empathy, eye contact*
- Was it genuinely open or were closed questions asked that limited the scope?
- What was the last thing the patient said?
- How the consultation is closed, including discussion of plans for the next review
- Any other relevant issues

For peer review only

Appendix 3

Consolidated criteria for reporting qualitative studies (COREQ): 32-item checklist

1.	Interviewer/facilitator	The interviews, focus groups and observations were conducted by Cindy Mann, with the exception of 5 observations and one interview that were carried out by Polly Duncan.
2.	Credentials	Cindy Mann had an MSc and previous qualitative research experience at the time of the study. Polly Duncan is an academic GP and was gaining qualitative research experience at that time.
3.	Occupation	CM was a senior research associate, Polly Duncan was a GP with an academic training fellowship
4.	Gender	Both female
5.	Experience and Training	CM has training and over 5 years experience in qualitative research and research methods. Experience in various environments (primary care and secondary care) as a researcher, research nurse and clinical nurse and experience as a counsellor and group facilitator. PD is a qualified GP with additional academic experience of research.
6.	Relationship established	Prior to study commencement, the interviewer and the participants had no previous contacts. Rapport was built before interview, focus groups or observations by answering questions from participants and taking informed consent.
7.	Participant knowledge of the interviewer	The participants did not have prior knowledge of the interviewer before the study. When participants were recruited, they were provided with an information leaflet about the study and purpose of the interview/focus group/observation which was repeated prior to data collection beginning. Information about the researcher was not provided other than her role in the research team.
8.	Interviewer characteristics	The principle qualitative researcher (CM) is a white, university-educated British woman with nursing, counselling and research qualifications. Qualitative research is always influenced by the perspective of the researcher, and in this case the nursing perspective and

For peer review only

	primary care clinical experience may have fed into the way some clinical participants were interviewed.
9. Methodological orientation and Theory	The key methodological framework used was a framework for process evaluation for cluster randomised trials and the MRC guidance for the process evaluation for complex interventions framework. Mixed methods were used, and thematic analysis was used for the qualitative data.
10. Sample	Intervention practices taking part in the 3D trial were purposively sampled for the process evaluation based on their characteristics. Individual staff members and clinicians of those practices that agreed to take part in the process evaluation were separately invited to take part in the process evaluation, based on their roles. Patient participants were sampled based on their responses to a baseline questionnaire.
11. Method of approach	Patient participants were approached by invitation letter including information sheet and staff and clinicians by email with invitation letter and information attached. In both cases follow up contact was made to discuss possible participation and to arrange the details.
12. Sample Size	The total number of interviews with staff, including informal debriefs after 3D reviews, was 32 (18 GPs, 20 nurses and 9 administrator interviews). Some individuals were interviewed twice so the actual number of those interviewed was 11 GPs, 14 nurses, 7 administrators and 38 patients (including the 22 patients who attended a focus group). 28 intervention review observations were carried out.
13. Non-participation	Some patients refused interviews or focus group and 1 nurse refused review observation
14. Setting of Data Collection	Interviews were conducted in GP practices, patients' homes or, in the case of focus groups, local halls, depending on convenience and patient preference. Observations were all carried out at the GP practice.
15. Presence of non-participants	Patients' carers were sometimes present at review observations, interviews or focus groups but all of them also provided consent. The researcher was present in a non-participatory role at observations

For peer review only

16. Description of the sample	GPs, administrators, practice nurses and patients from 9 different GP practices
17. Interview guide	Interview guides, a focus group schedule and an observation guide were used to act as a checklist but without imposing a set structure
18. Repeat interviews	Repeat interviews were carried out with some nurses, GPs and administrators who were interviewed both at beginning and end of the trial
19. Audio-/visual recording	We used audio recording to collect all data.
20. Field notes	Field notes were made during the observations to note participant expression, or other non-verbal cues and in all instances of data collection to describe the ambience of the GP practice and reception and aspects of the environment and interaction.
21. Duration	Pre-arranged interviews lasted 15-50 minutes and follow-up interviews lasted 5-24 minutes. Focus groups lasted an hour. Review observations lasted between 20 and 60 minutes.
22. Data Saturation	The concept of information power was used, rather than data saturation, since it is more in keeping with the process evaluation focus.
23. Transcripts returned	Transcripts were not returned to participants for comment or correction.
24. Number of data coders	One (Cindy Mann), with double coding of a sub-sample by Alison Shaw, Lesley Wye, Polly Duncan and 2 members of the Patient Public Involvement group
25. Description of the coding tree	Not included in this manuscript because the purpose of this paper is not primarily to report the findings of a qualitative piece of research
26. Derivation of themes	Themes in the qualitative data were a priori based on intervention components, supplemented by themes identified in the data
27. Software	NVivo v11
28. Participant checking	No. Transcripts were not returned to the participants for checking.

For peer review only

29. Quotations presented	Yes, participant quotations are presented to illustrate the themes.
30. Data and findings consistent	Yes.
31. Clarity of major themes	Major themes are based around intervention components as the purpose of the paper is to assess implementation fidelity
32. Clarity of minor themes	Not applicable

For peer review only

Appendix 4: Electronic monitoring of review component delivery

Practice	Penn	Priestman	Sharples	McReady	Harvey	Blackwell	Guppy	Lovell	Tothill	Beddoes	Dunbar	Plimsoll[1]	Carpenter	Davy	Cabot	Martineau	ALL
3d agenda printed	97%	92%	100%	89%	97%	81%	95%	98%	98%	100%	100%	70%	97%	58%	100%	92%	96%
3d health plan printed	77%	81%	97%	91%	62%	31%	23%	100%	80%	98%	85%	39%	85%	80%	98%	67%	83%
adherence meds	95%	61%	94%	96%	65%	92%	63%	100%	39%	67%	62%	44%	54%	50%	93%	64%	71%
EQ5D pain	47%	97%	100%	71%	100%	96%	65%	52%	100%	98%	100%	5%	100%	100%	100%	95%	83%
GP first goal noted	100%	97%	100%	100%	76%	96%	100%	100%	102%	98%	102%	44%	100%	95%	93%	97%	94%
Most important problem on nurse view	100%	97%	100%	100%	100%	96%	100%	100%	100%	100%	100%	100%	100%	100%	100%	97%	99%
Pharmacist comment	84%	100%	95%	82%	105%	78%	83%	100%	107%	106%	100%	38%	78%	43%	100%	102%	88%
Pharmacist comments noted?		56%	53%		47%	77%			68%	69%	92%		56%	80%	95%	64%	69%
PHQ9 done	97%	97%	100%	91%	91%	96%	98%	100%	98%	98%	100%	100%	97%	94%	100%	103%	98%
what GP can do about main problem	92%	89%	100%	98%	71%	73%	98%	76%	100%	89%	102%	33%	87%	80%	90%	72%	84%
what patient can do about main problem noted	77%	92%	86%	96%	76%	73%	100%	91%	100%	93%	100%	39%	97%	85%	90%	78%	86%
3D participants Pharmacist comment	84%	100%	95%	82%	105%	78%	83%	100%	107%	106%	100%	38%		43%	100%	102%	88%

Key: Range of fidelity from red (worst) to green (best)
Grey-shaded column headers indicate case study practices
Practice ID: 60 = Harvey; 46 = Lovell; 26 = Beddoes; 69 = Davy
Some values are greater than 100% because percentages were calculated based on the number of participants remaining in the trial at the end
[1]This practice stopped delivering the intervention and withdrew from the process evaluation
